# Towards large-scale chemical reaction image parsing *via* a multimodal large language model

**DOI:** 10.1039/d5sc04173b

**Published:** 2025-10-07

**Authors:** Yufan Chen, Ching Ting Leung, Jianwei Sun, Yong Huang, Linyan Li, Hao Chen, Hanyu Gao

**Affiliations:** a Department of Chemical and Biological Engineering, Hong Kong University of Science and Technology Hong Kong SAR China hanyugao@ust.hk; b Department of Chemistry, Hong Kong University of Science and Technology Hong Kong SAR China; c Department of Data Science, City University of Hong Kong Hong Kong SAR China; d Department of Computer Science and Engineering, Hong Kong University of Science and Technology Hong Kong SAR China

## Abstract

Artificial intelligence (AI) has demonstrated significant promise in advancing organic chemistry research; however, its effectiveness depends on the availability of high-quality chemical reaction data. Currently, most published chemical reactions are not available in machine-readable form, limiting the broader application of AI in this field. The extraction of published chemical reactions into structured databases still relies heavily on manual curation, and robust automatic parsing of chemical reaction images into machine-readable data remains a significant challenge. To address this, we introduce the Reaction Image Multimodal large language model (RxnIM), the first multimodal large language model specifically designed to parse chemical reaction images into machine-readable reaction data. RxnIM not only extracts key chemical components from reaction images but also interprets the textual content that describes reaction conditions. Together with a specially designed large-scale dataset generation method to support model training, our approach achieves excellent performance, with an average *F*_1_ score of 88% on various benchmarks, surpassing state-of-the-art methods by an average of 5%. This represents a crucial step toward the automatic construction of large databases of machine-readable reaction data parsed from images in the chemistry literature, providing essential data resources for AI research in chemistry. The source code, model checkpoints, and datasets developed in this work are released under permissive licenses.

## Abstract 2

RxnIM, the first MLLM for parsing chemical reaction images into machine-readable data. Trained on large synthetic and real data, it attains 88% *F*_1_, ∼5% over SOTA, facilitating the construction of large-scale chemical databases for downstream tasks.

## Graphical abstract

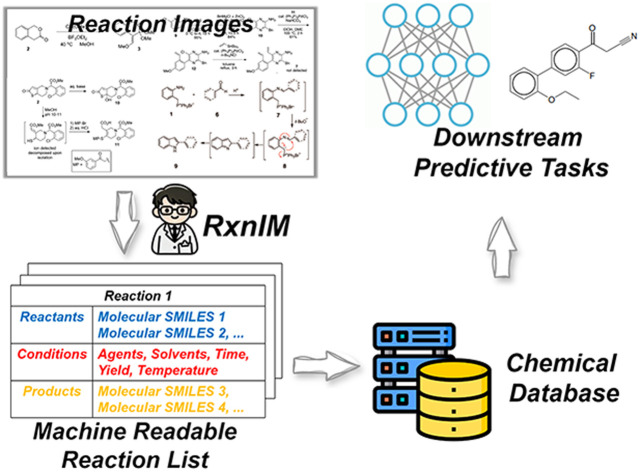

## 1 Introduction

The field of organic chemistry has witnessed a transformative shift with machine learning techniques, enabling significant advancements in retrosynthesis, reaction prediction, and condition recommendation. As researchers increasingly leverage these methodologies to explore complex chemical phenomena, high-quality machine-readable chemical reaction data is essential. Despite the wealth of chemical knowledge documented in the literature, the data required for effective machine learning applications remains largely fragmented and predominantly inaccessible in a format suitable for computational analysis.^1–5^

Traditional approaches to chemical reaction data extraction are predominantly manual, involving labor-intensive curation processes that are susceptible to human error and inefficiencies. This reliance on manual extraction limits the scalability of data acquisition as well as the potential for comprehensive analysis across large datasets. There is an urgent need for automated solutions that can accurately and efficiently parse chemical reaction images, transforming them into structured data that can support advanced machine learning applications.

Substantial efforts have been devoted to automatic chemical reaction data extraction.^6–11^ For instance, the Pistachio dataset,^12^ primarily derived from patent text, utilizes a classic natural language processing pipeline that encompasses syntactic parsing and named entity recognition to identify chemical names,^13,14^ followed by event extraction to organize these chemicals into reactions. To handle the varied text found in journal articles, Guo *et al.* developed a deep learning approach^15^ that breaks down the task into product extraction and reaction role labeling, utilizing sequence tagging techniques based on pre-trained language models. Zhong *et al.* proposed a reaction extraction system based on large language models (LLMs),^16^ which utilized the natural language understanding ability of LLMs, and expanded the scope of existing predefined reaction roles to include important attributes that have been ignored before, thus providing a more comprehensive and accurate description of chemical reactions. Despite these advancements, existing work primarily focuses only on processing textual information.

Images serve as a more intuitive medium for documenting chemical reactions, providing clear visualization of molecular structures and the logical flow of multi-component and multi-step reactions. Yet, reaction image parsing remains under-explored, largely due to the complexity of reaction images and the variability of drawing styles. Previous works have attempted to detect the location of molecular objects from images^3,4^ or recognizing their chemical structures.^17–20^ These methods often struggle to understand the role of different components and their logical connections, which are critical for a comprehensive understanding of the chemical reactions depicted.^3,4^ For reaction image parsing, Qian *et al.* developed a single encoder-decoder model^5^ closely following Pix2Seq,^21^ attempting to parse reaction data from images directly *via* an image-to-sequence translation. This approach demonstrated considerable promise, yet it still frequently failed to parse data from images of more complicated reaction patterns. Additionally, the multimodality of reaction information has not been explicitly addressed in previous methods. For example, for the text of reaction conditions and other auxiliary information, existing methods typically rely on external optical character recognition (OCR) tools to recognize the characters, and do not further process the information (*e.g.*, whether the text describes the agents, solvents, time, temperature, or yield), resulting in less comprehensive final parsed data.

Recently, as an essential subset of LLMs,^22–29^ multimodal large language models (MLLMs) represent a significant breakthrough in computer vision.^30–42^ They have demonstrated impressive capabilities in both traditional visual tasks, such as object detection and instance segmentation, as well as in more complex tasks like referring expression comprehension and generation.^43^ Moreover, MLLMs have shown OCR capabilities in some text-related visual tasks in chemistry with prompt engineering.^44,45^ Therefore, MLLMs present a promising solution for parsing data from chemical reaction images, an area that remains largely uncharted in the literature. In this context, we investigate whether a single unified MLLM can jointly localize reaction components and interpret condition texts with chemical-grade accuracy within complex reaction images. We further hypothesize that task-driven cross-modal instructions and model architecture will resolve overlapping graphical elements and textual annotations more reliably than separate vision and text pipelines, and that the visual reasoning capabilities of such an MLLM will substantially outperform traditional vision-only or pipeline-based approaches across diverse layout conventions.

**Fig. 1:**
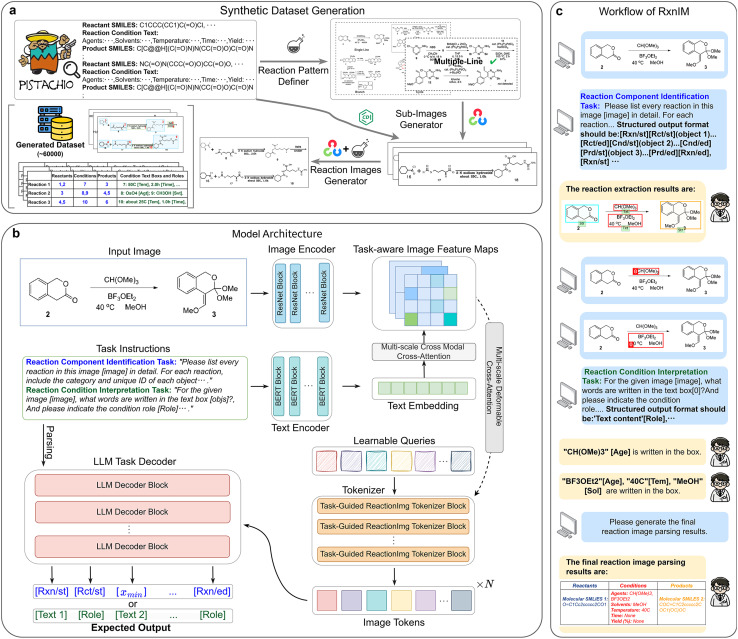
Dataset generation and overview of the proposed RxnIM. (a) Synthetic dataset generation pipeline. We obtain textual reaction information in the Pistachio dataset, generate visual reaction components, and create sub-images based on predefined reaction patterns. These sub-images are combined to form the final synthetic reaction image. This process resulted in the creation of a large-scale chemical reaction image parsing dataset containing 60 000 diverse images. (b) Model architecture of our RxnIM. The model incorporates four key components: (1) a unified task instruction for standardizing chemical reaction image parsing tasks, (2) a multimodal encoder that aligns image information with task instructions, (3) a ReactionImg tokenizer to convert image features into tokens, and (4) an open-ended LLM decoder that generates the final output. (c) Workflow for chemical reaction image parsing using RxnIM, where results from two tasks are combined and molecular structures are converted into machine-readable formats like SMILES or Molfile.

In this paper, we present Reaction Image parsing Multimodal large language model (RxnIM), the first multimodal large language model specifically designed for parsing chemical reaction images. We first created a large-scale synthetic dataset (Fig. 1(a)) by a novel data generation algorithm that extracts textual reaction information from the Pistachio dataset, generates visual reaction components, and assembles sub-images according to predefined reaction patterns. Then we designed the RxnIM architecture (Fig. 1(b)), which integrates a unified task instruction framework, a multimodal encoder to align image features with text-based instructions, a ReactionImg tokenizer for converting visual features into tokens, and an open-ended LLM decoder for generating the parsing output. We trained RxnIM using a three-stage training strategy with a unified language-based task instruction for different chemical reaction image parsing tasks. The first stage was pretraining the model's object detection capability on the large-scale synthetic dataset. In the second stage, the model was trained to identify the reaction components and extract reaction conditions using the synthetic dataset. In the final stage, the model was fine-tuned on the synthetic dataset with a smaller, manually curated dataset to enhance its performance on real reaction images. Finally, we apply a straightforward and comprehensive workflow using RxnIM (Fig. 1(c)) to seamlessly identify reaction components, extract reaction conditions, and convert molecular structures into machine-readable formats such as SMILES or Molfile. Further details can be found in the SI Materials and methods section. Unlike previous rule-based^3,4^ or single-task models,^5^ RxnIM comprehensively understands chemical reaction images, flexibly integrates multiple parsing tasks, and produces more accurate and holistic outputs.

The model achieved an average *F*_1_ of 88% (soft match score, defined in SI Materials and methods section) across various benchmarks for the reaction component identification task, significantly outperforming the state-of-the-art method by an average of 5%. Additionally, our tests highlighted the model's superior abilities in interpreting textual information that describes reaction conditions. We further developed a web application that can easily be used and deployed. The web application is hosted at https://huggingface.co/spaces/CYF200127/RxnIM. Since RxnIM was trained on promising large-scale data and offered as a ready-to-use open-source tool, we believe it will greatly reduce the workload and enable the construction of high-quality datasets for the research community and promote machine-learning-driven innovations in organic chemistry.

## 2 Results and discussion

In our workflow, the chemical reaction image parsing task is divided into two sub-tasks: reaction component identification and reaction condition interpretation. The reaction component identification task involves identifying all the reactions, segmenting their components, and understanding their roles (such as reactant, condition, or product) in a reaction image. The reaction condition interpretation task is to extract the detailed condition in a reaction by recognizing the words in the text regions that describe reaction conditions and understanding their meanings (*e.g.*, names of agents or solvents, temperature, time, and yield). Further details of the task design can be found in the SI Materials and methods section.

To minimize the effort for data labeling, we primarily used “synthetic data” to train our models. Specifically, we use structured reaction data from a large-scale chemical reaction database, Pistachio,^12^ to construct images of chemical reactions following the general rules and styles in the chemical literature. For example, for a single-step reaction, we first draw the images of reactant and product molecular structures using cheminformatics tools, and then place them on a canvas and draw an arrow that points from the reactant to the product. Agent information is placed above the arrow, while solvent, temperature, reaction time and yield are placed below the arrow. In this way, we can construct a large number of labeled images of chemical reactions automatically. To account for the complexity of real chemical reaction images, we performed data augmentation in font size, line width, size of the molecular images, and reaction pattern (*e.g.*, single-line, multiple-line, branch, and cycle). Full descriptions of the image generation and examples of generated images are available in the SI Materials and methods section. Using this approach, we generated 60 200 synthetic images along with their corresponding ground truth data as our primary dataset. For each image, ground truth data includes the positions and roles of the reaction components, as well as the reaction condition texts. We divided the data into training, validation, and test sets using an 8 : 1 : 1 ratio. To ensure the model performs well on a broader range of reaction images found in real literature, we also incorporated a small-scale real reaction image dataset manually labeled by Qian *et al.*^5^ We followed the original split of the real data set and used it in both the training and testing phases for the reaction component identification task. The details of each dataset can be found in SI Additional Note 1.

The training process was conducted in three stages, each utilizing different datasets and tasks. In the first stage, the model was trained on the synthetic dataset, focusing on the object detection task to accurately locate objects within the reaction images. In the second stage, the model was further trained on the synthetic dataset, incorporating both the reaction component identification and reaction condition interpretation tasks to enhance its ability to understand the roles and contents of the parsed objects. In the final stage, the model was fine-tuned using the real reaction image dataset specifically for the reaction component identification, allowing it to adapt to the more diverse and complex scenarios present in real-world chemical literature. The implementation details can be found in the SI Materials and methods section.

### 2.1 Performance on the reaction component identification task

For the reaction component identification task, we compared our RxnIM with current reaction component identification methods including rule-based OChemR^46^ and ReactionDataExtractor,^3^ ReactionDataExtractor 2.0 (ref. 4), learning-based models RxnScribe,^5^ as well as MLLMs such as Uni-Finder, Qwen2.5-Max,^40^ GPT-4o,^41^ and GPT-o3 (ref. 42) using evaluation metrics including precision, recall, and *F*_1_ score. We adopted the same concepts of “hard match” and “soft match” as described in the RxnScribe paper, where a hard match only counts instances where the prediction matches the ground truth exactly, while a soft match allows the labeling of the role of an agent as a reactant. The details of the evaluation metrics can be found in the SI Materials and methods section.

The results on the synthetic test dataset and the real test dataset are shown in Table 1. RxnIM demonstrates better performance on various metrics when compared with other methods. Specifically, in the soft match criteria, RxnIM achieves a precision of 91.6%, a recall of 90.8%, and an *F*_1_ score of 91.2% on the synthetic test dataset, outperforming the second best method, RxnScribe, by 4.0%, 6.8%, and 5.4%, respectively. On the real data set where the images are more diverse and complex, RxnIM still reports a precision of 86.9%, a recall of 82.8%, and an *F*_1_ score of 84.8%, outperforming the second best method by 3.1%, 6.3% and 4.8%, respectively. This indicates the advanced abilities of our model in extracting reactions in diverse reaction images, underscoring its robustness and adaptability to different levels of image complexity and variability. Under the hard match criteria, RxnIM reaches a precision of 86.4%, a recall of 85.9%, and a *F*_1_ score of 86.2% on the synthetic test dataset, outperforming the second best method by 7.9%, 10.3% and 9.1%, respectively, and a precision of 74.7%, a recall of 69.7%, and an *F*_1_ score of 72.1% on the real test dataset, surpassing the RxnScribe by 2.4%, 3.5% and 3.0%, respectively.

**Table 1:** Overall comparison of model performance on the reaction component identification task on different test datasets. We contrast the performance of RxnIM with other models on both synthetic and real datasets. We present detailed metrics for hard match and soft match criteria, including precision, recall, and *F*_1_ scores. Scores are all in %

Dataset	Model	Hard match			Soft match		
		Precision	Recall	*F*_1_	Precision	Recall	*F*_1_
Synthetic	OChemR^46^	8.1	7.1	7.8	15.9	12.8	14.2
	ReactionDataExtractor^3^	8.4	6.9	7.6	22.6	11.4	15.2
	ReactionDataExtractor 2.0 (ref. 4)	54.2	53.4	53.7	66.2	63.1	64.6
	RxnScribe^5^	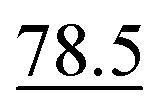	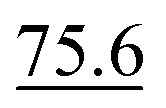	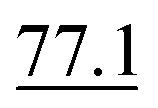	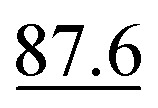	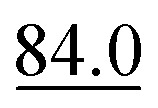	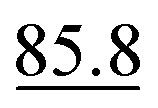
	Uni-Finder	4.0	3.1	3.5	5.6	4.5	5.0
	Qwen2.5-Max^40^	8.0	7.8	7.9	11.7	10.9	11.3
	GPT-4o^41^	17.1	16.1	16.6	20.3	19.4	19.8
	GPT-o3 (ref. 42)	55.6	55.1	55.3	61.2	60.4	60.8
	RxnIM	**86.4**	**85.9**	**86.2**	**91.6**	**90.8**	**91.2**
Real	OChemR^46^	4.4	2.8	3.4	12.4	7.9	9.6
	ReactionDataExtractor^3^	4.1	1.3	1.9	19.4	5.9	9.0
	ReactionDataExtractor 2.0 (ref. 4)	42.1	41.8	42.0	49.5	49.1	49.4
	RxnScribe^5^	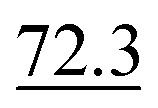	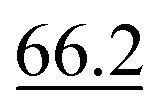	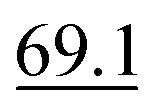	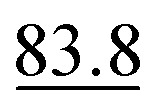	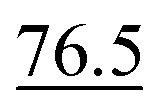	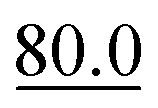
	Uni-Finder	3.1	2.9	3.0	4.1	4.0	4.0
	Qwen2.5-Max^4^	6.1	6.0	6.0	7.2	6.7	6.9
	GPT-4o^4^	12.1	11.2	11.6	14.6	14.2	14.4
	GPT-o3 (ref. 4)	44.1	43.4	43.7	51.1	51.8	51.4
	RxnIM	**74.7**	**69.7**	**72.1**	**86.9**	**82.8**	**84.8**

One of the main reasons for such improvement is that RxnIM refines the output sequence defined by RxnScribe by introducing paired start-end tokens for each reaction component, which provides more precise structural boundaries and reduces decoding ambiguity. Besides, our architecture also outperforms their traditional transformer encoder-decoder frameworks using multi-scale cross-modal attention and a task-guided tokenizer to capture text-augmented fine-grained visual details and generalize across related tasks, respectively. Furthermore, RxnIM is trained on a larger, purpose-built corpus of synthetic and real reaction images. Compared to RxnScribe, RxnIM consistently achieves higher precision, recall, and *F*_1_ score across all reaction categories. Overall, these advances make RxnIM a significant improvement over RxnScribe in both scope and performance.

It is notable that general multimodal large language models (Uni-Finder, Qwen2.5-Max, GPT-4o, and GPT-o3) exhibit inferior performance compared to RxnIM and specialized chemical models, especially under strict criteria (hard match). Uni-Finder is an MLLM specifically designed for information extraction from scientific literature, but it struggles with this task, which requires fine-grained image localization capability like other MLLMs. Among these general models, GPT-o3 performs relatively better, reaching 55.6% precision and 55.1% recall on synthetic data, and 44.1% precision and 43.4% recall on real data under the hard match criterion. GPT-o3 outperforms ReactionDataExtractor 2.0 on the real test set, which shows that its visual reasoning ability is more flexible and effective than the rule-based method in some complex cases. But it is still significantly behind RxnIM. This gap is indicative of the inherent limitation of generic MLLMs in handling highly specialized chemical image parsing tasks, underscoring the advantage of domain-specific training and model specialization as implemented in RxnIM. Similarly, under the soft match criteria, the general MLLMs still lag behind specialized models like RxnIM and RxnScribe, although the performance differences are somewhat reduced. This reflects the capability of general MLLMs to perform moderately well when exact labeling precision is relaxed, but also emphasizes the necessity of domain-tailored multimodal approaches like RxnIM to ensure high-quality chemical information extraction.

**Fig. 2:**
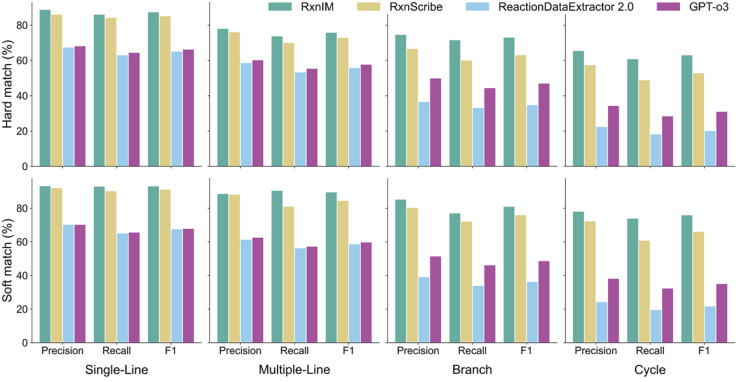
Comparison of model performance on the reaction component identification task on four different patterns of reaction images on the real test dataset. We display precision, recall, and *F*_1_ scores in hard match and soft match, of our model and current methods across four patterns of reaction images: single-line, multiple-line, branch, and cycle. The performance is evaluated to demonstrate the models' capabilities in accurately extracting reactions under varying image complexities and layouts.

We further broke down the performance comparison into four different patterns of reaction images on the real test dataset – (1) single line, where all reactions appear in the same line; (2) multiple line, where there are multiple lines of reactions, (3) branch, where branch is used when multiple reactions start from a common reactant, and (4) cycle, where multiple reactions are displayed in a cycle, in four best methods, RxnIM, RxnScribe, ReactionDataExtractor 2.0 and GPT-o3, shown in Fig. 2. A detailed data distribution for four reaction image patterns is shown in SI Additional Note 2 and Table S2. For single-line reaction images, RxnIM achieves a hard match *F*_1_ score of 87.3%, surpassing RxnScribe's score of 85.0%. In multiple-line images, RxnIM achieves a hard match *F*_1_ score of 75.7%, significantly outperforming RxnScribe's 72.8%. In branch images, RxnIM demonstrates a clear advantage, obtaining a hard match *F*_1_ score of 72.9%, compared to RxnScribe's 63.0%. For cycle images, RxnIM attains a hard match *F*_1_ score of 62.9%, notably higher than RxnScribe's 52.7% and significantly above GPT-o3's 30.9%. Under the soft match criterion, RxnIM consistently maintains superior performance, achieving scores of 93.0%, 89.4%, 80.8%, and 75.8% for single-line, multiple-line, branch, and cycle reaction images, respectively. RxnIM consistently outperforms RxnScribe and significantly exceeds the capabilities of ReactionDataExtractor 2.0 and GPT-o3 across all reaction image patterns, and the gap will be greater in more complex situations. Notably, GPT-o3, despite being a general multimodal large language model, shows relatively lower performance across all image patterns, with the largest performance gap in cycle patterns. This highlights the limitations of general MLLMs in specialized chemical tasks. Meanwhile, the performance of the rule-based method ReactionDataExtractor 2.0 remains at the lowest level. The superior performance of RxnIM, particularly in multiple-line, branch, and cycle patterns, underscores its enhanced capability for advanced image reasoning and localization, making it particularly suited to handling complex chemical reaction image scenarios. This result also provides users with an estimate of the reliability of model outputs based on the style and complexity of the reaction images that are being processed. Further discussions on the synthetic test dataset can be found in SI Additional Discussion 1 and Table S5.

**Fig. 3:**
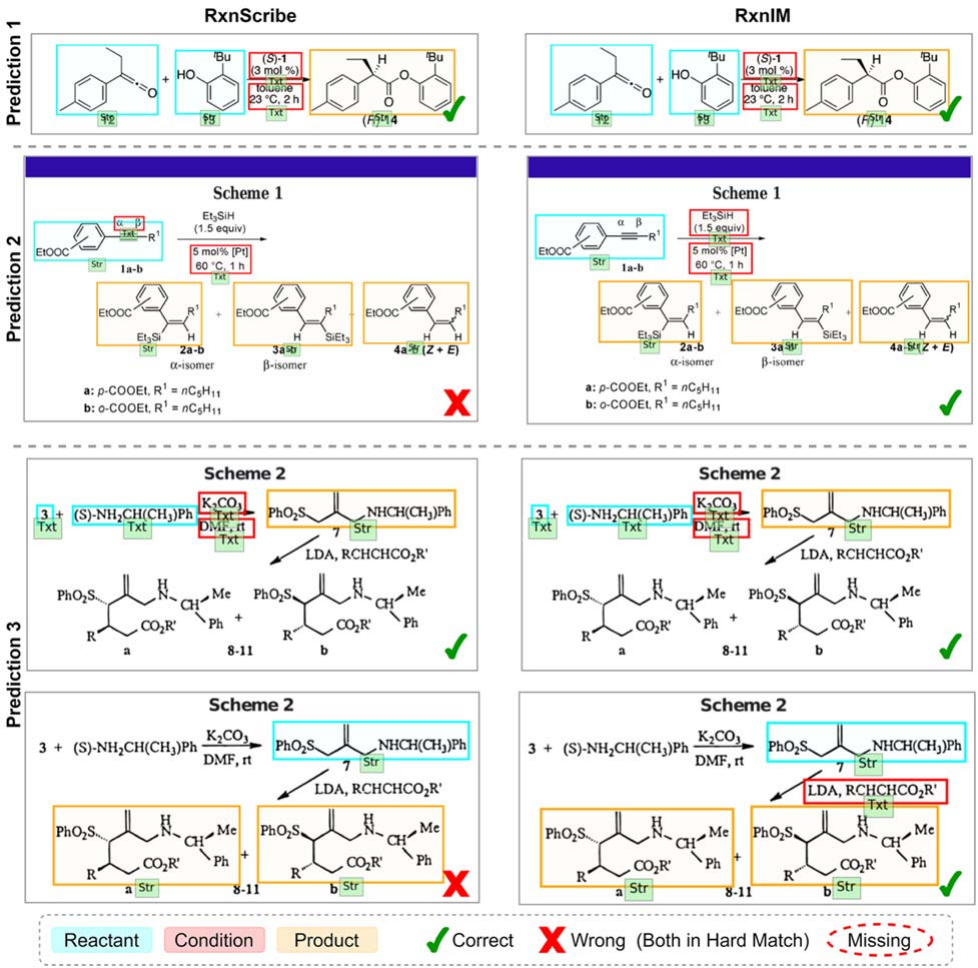
Visualization examples on the reaction component identification task compared to the current best method RxnScribe. We display the comparison between RxnScribe and RxnIM on the reaction component identification task across three different prediction examples. Each predicted reaction is visualized in a separate image, showing the predicted reaction components, including reactants, conditions, and products, with color-coded boxes representing different component types. Check marks and cross marks indicate correct and incorrect predictions, respectively, under the hard match criteria. The red dashed circle indicates that the reaction is not predicted. The DOI numbers of the relevant journal articles for these real reaction images can be found in SI Additional Note 3 and Table S3.

In addition to quantitative measures, we show some examples of the reaction component identification task in Fig. 3, compared to the second-best method, RxnScribe, to provide intuitive illustrations on the improvement achieved by RxnIM. RxnScribe and RxnIM both make the correct prediction in prediction 1, which is a simple single-line image. In prediction 2, RxnScribe misinterprets several key objects in reactants and conditions, leading to inaccuracies in the reaction representation. This is due to the diverse single-line image containing a complex molecular structure and being line-wrapped when placing products. For this example, RxnIM accurately depicts the relationship between molecules in different lines and clearly labels the relevant reaction conditions. In prediction 3, which is an unusual multiple-line image with a nonparallel arrow surrounded by condition text, RxnScribe makes mistakes in labeling conditions in the second reaction step. RxnIM, however, successfully decodes all reaction steps, showcasing its robustness in handling complex reaction images.

We further show some RxnIM's predictions on more complex reaction images in Fig. S2 to S7. Fig. S2 is a multiple-line image with four reactions. RxnIM correctly predicts all reactions, even when the products of the last reaction are placed vertically, which is a rare style. In Fig. S3, which is a branch image containing two different branches and three reactions in total, and RxnIM correctly identifies all reactions in each branch. Fig. S4 is a catalytic cycle that represents one of the most complex styles in reaction images. This image contains nine diverse reactions, with a blend of many different types of arrows, such as curved, branching, vertical, and bidirectional. RxnIM successfully makes eight correct predictions out of nine reactions. The only incorrect prediction is a reaction where the model misses a small-molecule byproduct, and the major reactants and products are well recognized. Fig. S5 shows a radially branching reaction scheme with condition texts placed at various angles and orientations rather than strictly horizontally. RxnIM accurately identifies all reaction components and correctly parses the angled condition texts, highlighting the model's robustness against layout variability. In Fig. S6, we illustrate RxnIM's ability to correctly interpret reaction arrows explicitly marked with a cross (×), indicating that these particular reaction paths do not occur or are otherwise invalid. The model successfully distinguishes these invalid reaction branches from the valid ones. Fig. S7 shows reactions involving reversible reactions denoted by bidirectional arrows. RxnIM accurately identifies both the forward and reverse directions, correctly interpreting the reversible nature of these chemical equilibria. These additional examples underscore RxnIM's comprehensive ability to handle various complex arrow notations and reaction layouts commonly encountered in chemical literature.

The performance of RxnIM across various metrics, datasets, and reaction image patterns underscores its effectiveness in handling the complexities of the reaction component identification task in the chemical literature, highlighting its exceptional image reasoning and localization abilities to extract every reaction step in complex and diverse reaction images.

### 2.2 Performance on the reaction condition interpretation task

**Fig. 4:**
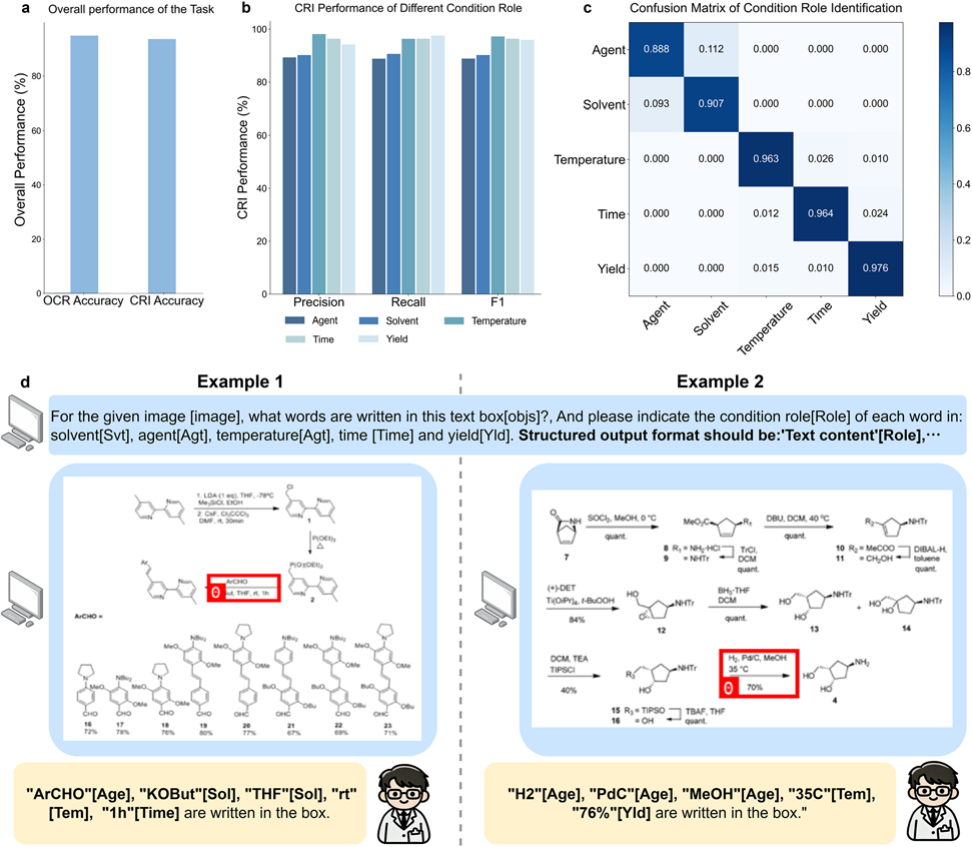
Performance and visualization examples on the reaction condition interpretation task. (a) Overall performance on the reaction condition interpretation task in OCR and CRI (Condition Role Identification) accuracy. (b) The CRI performance in precision, recall, and *F*_1_ scores on five different condition roles: agent, solvent, temperature, time, and yield. (c) The confusion matrix detailing the model's performance in correctly identifying these condition roles, highlighting areas of accurate and confused classifications. (d) Example model outputs for the reaction condition interpretation task across two different predictions. Each prediction involves extracting and identifying the text and corresponding roles within reaction condition boxes. The output format indicates the recognized text along with its assigned condition role, such as agent [Age], solvent [Sol], temperature [Tem], time [Time], and yield [Yld].

RxnIM uniquely leverages the multimodal capabilities of LLMs to perform multiple reaction image parsing tasks within a unified framework. By integrating visual and textual information, our model is able to not only identify the locations of reaction components but also interpret the textual content in the components, *i.e.*, perform the reaction condition interpretation task, differentiating it from existing models. The overall performance of RxnIM on the reaction condition interpretation task is outlined in Fig. 4(a). We evaluated the performance from two perspectives: (1) whether the text of the reaction condition is correctly recognized, which we term “condition OCR”, and (2) whether the roles of different elements in the reaction condition are correctly understood, which we term “condition role identification (CRI)”. For condition OCR, RxnIM achieved a high accuracy of 94.9%, indicating the model's effectiveness in recognizing and converting text within chemical images into editable and searchable data.

For the CRI task, the model reached an accuracy of 93.6%. The CRI performance in Fig. 4(b) and the confusion matrix in Fig. 4(c) further detail the model's performance across various condition roles such as agent, solvent, temperature, time, and yield. The model demonstrates strong performance in identifying agents with a precision of 89.3%, a recall of 88.8%, and an *F*_1_ score of 89.1%. For solvent, the precision is 90.2%, recall is 90.7%, and *F*_1_ score is 90.4%. These results suggest that the model effectively distinguishes between chemical agents and solvents. It is worth noting from the confusion matrix that agents and solvents are more often misidentified with each other, as compared to with other elements. This is likely due to some inherent ambiguity of these two roles – some chemicals can both serve as the solvent to dissolve the reactants, and the agent to promote reactivity. For numerical parameters like temperature, time, and yield, the model exhibits high accuracy. The precision, recall and *F*_1_ scores are 96.3%, 96.4%, and 97.6%, respectively, indicating strong performance in identifying and classifying these crucial elements in reaction conditions. The confusion matrix further confirms the model's accuracy, showing minimal misclassification among these categories.

We further compare the accuracy of our model with the MLLMs mentioned in the previous section on five condition roles in Table 2, while other methods cannot classify the roles. We asked these models to find all reaction conditions from a reaction image and classify them. RxnIM consistently achieves the highest accuracy across all roles, significantly outperforming general MLLMs. Notably, RxnIM achieves an accuracy of 88.9% for agents and 90.1% for solvents, clearly surpassing GPT-o3, the second-best model, which scores 85.3% and 87.1%, respectively. It is noteworthy that general MLLMs perform relatively poorly in accurately identifying agents and solvents but achieve comparatively higher accuracy for numerical parameters such as temperature, time, and yield. For these parameters, RxnIM further demonstrates superior performance, achieving accuracies of 96.3%, 96.4%, and 97.6%, respectively, indicating substantial improvement over general MLLMs and highlighting its specialized capability in accurately extracting and classifying detailed chemical reaction conditions. For numerical parameters like temperature, time, and yield, the model exhibits high accuracy. The precision, recall, and *F*_1_ scores are 96.3%, 96.4%, and 97.6%, respectively, indicating strong performance in identifying and classifying these crucial elements in reaction conditions. The confusion matrix further confirms the model's accuracy, showing minimal misclassification among these categories.

**Table 2:** Per-role CRI accuracy on the reaction condition interpretation task. We report the accuracy (%) of RxnIM, Uni-Finder, Qwen2.5-Max, GPT-4o and GPT-o3 across the five condition roles: agent, solvent, temperature, time and yield

Model	Agent	Solvent	Temperature	Time	Yield
Uni-Finder	81.2	80.1	91.5	92.4	91.3
Qwen2.5-Max^40^	77.2	79.4	90.4	91.2	91.5
GPT-4o^41^	81.5	83.0	93.2	94.1	95.1
GPT-o3 (ref. 42)	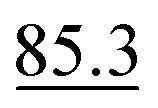	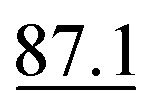	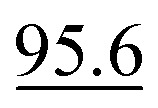	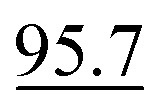	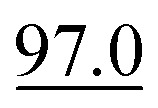
RxnIM	**88.9**	**90.1**	**96.3**	**96.4**	**97.6**

We visualize some examples of this task in Fig. 4(d). RxnIM exhibits strong performance in accurately extracting and categorizing text within reaction conditions. In two separate predictions, it effectively identifies and assigns roles to chemical agents, solvents, temperatures, and times. For instance, in the first example, it correctly labels ‘ArCHO’ as an agent and ‘THF’ as a solvent, while in the second, it accurately recognizes ‘H2’ and ‘PdC’ as agents, and ‘35C’ as the temperature. These results underscore the model's precision and utility in parsing condition text information in reaction images, making the final reaction component identification results more detailed and comprehensive. We further show more examples in Fig. S8 to demonstrate RxnIM's ability to capture condition texts that fall outside the five predefined roles and correctly omit role tokens. In example 1, RxnIM accurately extracts the tilted, non-horizontal text “R1 = Ar, Alk”, “R2 = Ar, Alk” and “R3 = CO2Alk” without assigning any role token. In example 2, it extracts “MeOH” and “pH 10–11” and recognizes that “pH” is not among the core categories, refraining from outputting a role token. These cases highlight RxnIM's flexibility in handling unexpected or auxiliary condition text while maintaining clean, structured output.

Overall, our comprehensive evaluation of RxnIM shows that it consistently outperforms the current methods. It demonstrates promising abilities in image reasoning, localization, and OCR, proving to be a reliable tool for chemical reaction image parsing and machine-readable reaction database construction.

### 2.3 Reaction SMILES output using RxnIM.web

RxnIM.web is a web application that combines the outputs of the previously described tasks using the proposed workflow for reaction image parsing (Fig. 1(c) and SI Materials and methods section). We also provide an example of RxnIM.web in action in Fig. S9. In this case, the RxnIM.web begins with the user uploading a multiple-line reaction image. RxnIM.web will first run the reaction component identification task to extract the regions of reactants, conditions, and products for each reaction. Visualized outputs are presented in the Reaction extraction output panel. Each reaction is displayed individually in an image. Then, RxnIM.web will run reaction condition interpretation tasks for each condition text region to extract the detailed conditions for each reaction. Molecular objects are then processed by our previously proposed molecular recognition model MolNexTR^47^ to obtain machine-readable SMILES strings. Finally, RxnIM.web will integrate this information in the Reaction image parsing output panel. Additionally, in the Machine-readable data output panel, we integrate the SMILES part of each reaction in the reaction image parsing output into reaction SMILES strings, making them accessible for common chemistry tools such as RDKit and ChemDraw. Each reaction structure is also visualized as a 2D molecular diagram by RDKit. Besides, the reaction image parsing output can also be downloaded in a machine-readable JSON file format in this panel.

**Fig. 5:**
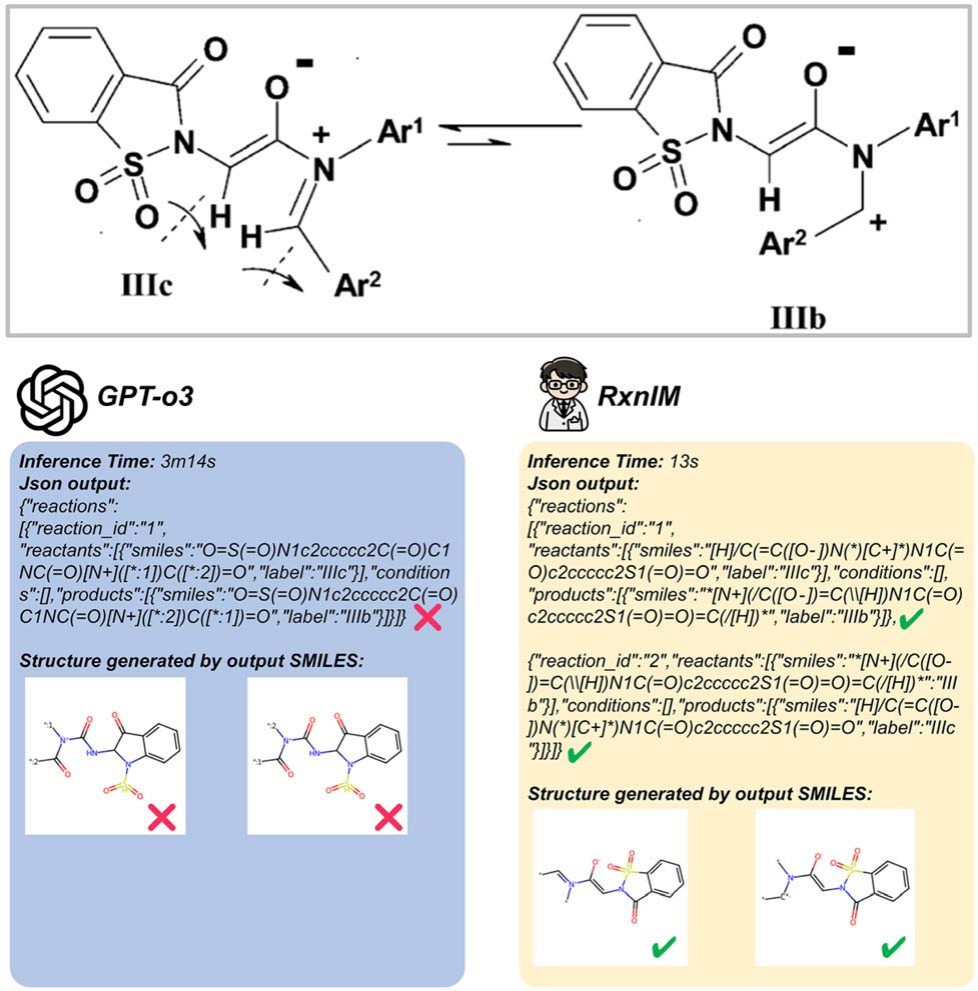
Visualization examples of the final JSON output compared to GPT-o3. This example compares RxnIM and GPT-o3 on a reversible reaction image. RxnIM completes inference in 13 s, correctly outputs both reaction directions in JSON with accurate SMILES including aromaticity and formal charges. In contrast, GPT-o3 requires over 3 min, returns only one reaction direction with incorrect SMILES, and fails to capture the correct chemical structure.

Since our synthetic datasets were generated from the Pistachio database, exact ground-truth reaction SMILES annotations were readily available. However, our real datasets contain only bounding-box annotations without corresponding reaction SMILES. Thus, we evaluate reaction SMILES exact match accuracy using only the synthetic dataset. We compare our model to the MLLMs mentioned in the previous section. We let these models directly output SMILES following a predefined data structure. As shown in Table 3, RxnIM significantly outperforms all MLLMs, achieving a reaction SMILES exact-match accuracy of 80.2%, compared to GPT-o3's 32.7%, GPT-4o's 18.7%, Uni-Finder's 12.3%, and Qwen2.5-Max's 9.6%. Furthermore, RxnIM exhibits substantial inference-time advantages, requiring only 17 seconds per image compared to significantly longer times by other models, such as GPT-o3 (2 minutes 45 seconds) and GPT-4o (47 seconds). We provide a visualized example in Fig. 5, comparing RxnIM with GPT-o3 on a challenging case involving a real reversible reaction image indicated by a bidirectional arrow and containing aromaticity and formal charge. In this example, RxnIM correctly recognizes and outputs both reaction directions separately, accurately generating correct SMILES strings that include aromaticity and formal charges, demonstrating its robust chemical feature extraction capability. In contrast, GPT-o3, despite significantly longer inference time (3 minutes 14 seconds compared to RxnIM's 13 seconds), incorrectly outputs only one direction of the reaction, and the SMILES strings generated are chemically incorrect, missing critical details related to aromaticity and formal charges.

**Table 3:** Reaction SMILES exact match accuracy and average inference time per image in the final output. We report the reaction SMILES exact match accuracy (%) of RxnIM, Uni-Finder, Qwen2.5-Max, GPT-4o and GPT-o3 on the synthetic datasets and the average inference time per image

Model	Inference time per image	Exact match accuracy
Uni-Finder	1 m 4 s	12.3
Qwen2.5-Max^40^	50 s	9.6
GPT-4o^41^	47 s	18.7
GPT-o3 (ref. 42)	2 m 45 s	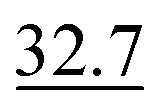
RxnIM	17 s	**80.2**

To further assess and mitigate prediction errors, we conducted a systematic error analysis (see SI Additional Discussion 2 and Fig. S11), which indicates that the predominant errors arise from molecular recognition inaccuracies when converting molecular structures into SMILES. This type of error can be reduced with future improvements of the molecular image recognition models. A secondary source of errors stems from highly complex reaction layouts, where the model may fail to fully capture the relationships among reactants and products or may omit certain reaction steps. In addition, a comparatively minor proportion of errors is attributable to condition interpretation, where textual information is misclassified. In practice, we integrate a Human-AI collaboration workflow into RxnIM.web (see SI Section 1.8), which allows chemists to visually compare parsed reactions with the original image and rapidly correct misclassifications. Overall, these results demonstrate the strength and efficiency of RxnIM in extracting chemically meaningful and precise molecular information, highlighting both its practical value for downstream cheminformatics applications and the importance of complementary workflows in constructing reliable datasets.

### 2.4 Effect of model components and configurations

**Fig. 6:**
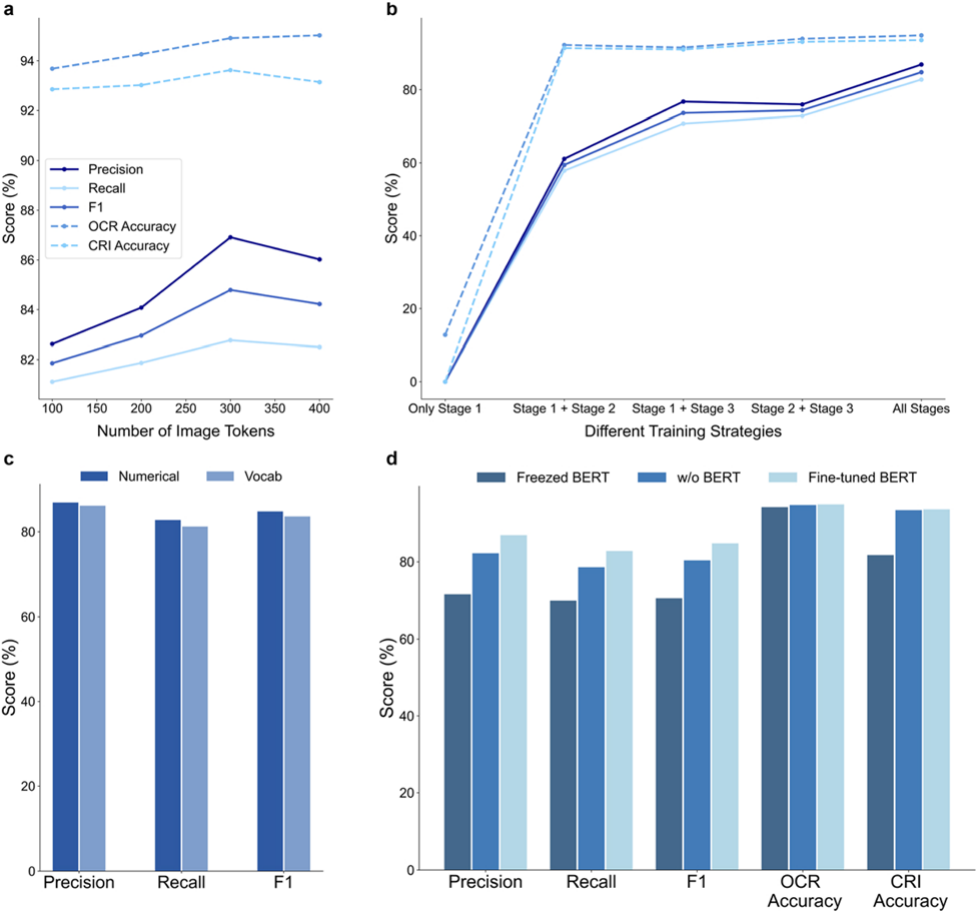
Model performance with different components and configurations (a) effect of varying the number of image tokens in precision, recall, *F*_1_ score, OCR accuracy, and CRI accuracy. (b) Influence of training strategies using different training stages, showing performance improvements across multiple training stages. (c) Compares the use of special location tokens *versus* numerical tokens in position representation. (d) Effect of the text encoder, contrasting performance with a frozen BERT, without BERT, and with a fine-tuned BERT. More discussions of model performance with different components and configurations are illustrated in SI Additional Discussions 3 and 4.

In this section, we provide evaluations of the components and configurations that influence the performance of our model in terms of precision, recall, and *F*_1_ under the soft match criteria for the reaction component identification task, as well as OCR accuracy and CRI accuracy. All evaluations for the reaction component identification task are conducted on the real test dataset. The results are shown in Fig. 6.

#### 2.4.1 Number of image tokens

In our exploration of the effect of varying the number of image tokens, depicted in Fig. 6(a), we observe a clear trend of increasing performance across all metrics as the number of tokens increases from 100 to 300. The performance peaks at 300 tokens, achieving a soft match *F*_1_ score of 84.8%, OCR accuracy of 94.9%, and CRI accuracy of 93.6%. This improvement suggests that a higher number of tokens allows the model to capture more detailed information from the images, enhancing its ability to differentiate and recognize complex structures and reaction components. However, as the number of tokens exceeds 300, we observe a slight decline in performance at 400 tokens. This decrease may be due to the reduced effectiveness of capturing long-range dependencies between tokens, as more tokens could represent smaller image regions, leading to diminished global context understanding. This suggests that 300 tokens provide an optimal balance, capturing sufficient detail while maintaining effective long-range relationships. Therefore, we select 300 image tokens as the ideal configuration to maximize performance without compromising the model's ability to understand the overall image structure.

#### 2.4.2 Influence of training strategies

The analysis of training strategies using different training stages is shown in Fig. 6(b). Detailed training strategies are described in the SI Materials and methods section and SI Additional Notes 4.

When using only Stage 1, the model cannot effectively perform the two downstream tasks because this stage focuses solely on pre-training the vision backbone for object detection. Once Stage 2 is incorporated (Stage 1 + 2), the performance increases substantially, highlighting the importance of the reaction component identification and reaction condition interpretation tasks in enabling cross-modal reasoning.

For the mixed strategies, Stage 1 + 3 and Stage 2 + 3 also lead to performance improvements over single-stage training. However, both settings converge to slightly lower scores compared with the full three-stage pipeline, suggesting that skipping any intermediate stage results in suboptimal learning. In particular, Stage 1 + 3 benefits from visual pre-training and real-data fine-tuning but misses the multi-task training that builds alignment between image and text features; Stage 2 + 3 benefits from multi-task training and real-data adaptation but lacks the foundation provided by object detection pre-training.

The all stages pipeline consistently achieves the best performance, demonstrating the cumulative benefits of our sequential strategy. These results confirm that each stage plays a distinct and complementary role: Stage 1 establishes localization ability, Stage 2 enhances recognition and semantic parsing, and Stage 3 adapts the model to real-world data. Together, they ensure both strong performance on synthetic benchmarks and robust generalization to practical applications.

#### 2.4.3 Special location tokens *vs.* numerical tokens

We compared the different position representation techniques in Fig. 6(c). Detailed position representation is described in the SI Materials and methods section. The model using numerical representations outperforms the one using vocabulary-based representations. This advantage likely arises from the numerical method's ability to provide more granular and precise positional information, which is critical in understanding spatial relationships in reaction images. Numerical tokens can encode exact coordinates and sizes, enabling the model to better differentiate between closely positioned elements and capture the detailed layout of reaction schemes. While numerical tokens might increase computational complexity during training, the trade-off is justified by the substantial improvement in model accuracy and reliability. This finding underscores the importance of precise positional representations in enhancing a model's image reasoning and localization capabilities.

#### 2.4.4 Influence of text encoder

We investigated the role of the text encoder BERT in the multimodal encoder in Fig. 6(d). The result illustrates that integrating BERT into the model without freezing any parameters provides a significant improvement in all metrics compared to configurations without BERT or with BERT and frozen parameters. This suggests that BERT's powerful contextual understanding of the task instructions significantly contributes to the model's performance, particularly when it is allowed to adapt to the specific context of the reaction parsing tasks. Freezing parameters while using BERT results in a noticeable drop in performance, particularly in CRI accuracy and soft match *F*_1_, underscoring the importance of dynamic parameter adjustment during training.

## 3 Conclusion

In this work, we introduced RxnIM, the first MLLM designed for reaction image parsing tasks. RxnIM demonstrates superior performance across diverse reaction images, particularly in handling complex cases, due to its robust image reasoning and localization capabilities. A large synthetic reaction image dataset and a structured three-stage training strategy significantly contributed to RxnIM's strong generalization and robustness in real-world applications. However, RxnIM currently faces limitations, including the inability to directly generate SMILES representations, difficulties in handling R-group substitutions, and challenges in linking reaction images with contextual text information such as footnotes or captions. Addressing these limitations through annotating more real-world complex reaction images, improving molecular recognition methods, developing more advanced table-to-template mapping algorithms, enhancing contextual understanding, and employing more sophisticated data generation strategies will further enhance RxnIM's applicability and reliability in cheminformatics. Overall, our work provides a powerful tool for chemical reaction image parsing while also serving as a valuable data resource for the wider research community. Additionally, it opens up new avenues for applying multimodal large-language models to broader image-based cheminformatics.

## Author contributions

YC wrote the main manuscript and developed the model and the synthetic data generation algorithm. YC and CTL prepared all figures and tables collaboratively. YC, HG, and CTL designed all experiments collaboratively. HG, HC, LL, JS, and YH supervised the work. All authors contributed to the manuscript. All authors read and approved the final manuscript.

## Conflicts of interest

There are no conflicts to declare.

## Supplementary Material

SC-016-D5SC04173B-s001

## Data Availability

All data and code used for this article are available in https://github.com/CYF2000127/RxnIM, which contains the annotated datasets and test and train and validation split. Intermediate files for each reaction data parsing task reported in this method are stored in this repository with the corresponding documentation. The model checkpoints can be downloaded directly from https://huggingface.co/datasets/CYF200127/RxnIM. An instance of the RxnIM web application can be accessed at https://huggingface.co/spaces/CYF200127/RxnIM. Supplementary information: full technical and experimental details. It includes our synthetic image generation pipeline based on the Pistachio dataset, task formulations using language-based instructions, architectural components of the RxnIM framework (multimodal encoder, task-guided tokenizer, LLM-based decoder), evaluation protocols, and detailed benchmarking results on both synthetic and real reaction image datasets. We also provide further detailed analyses, such as the impact of different base LLMs and image resolutions. See DOI: https://doi.org/10.1039/d5sc04173b.
