# Supplementary material for: Towards large-scale chemical reaction image parsing via a multimodal large language model
Source: Chem Sci. 2025 Oct 7;16(45):21464–74. doi: 10.1039/d5sc04173b (PMC12522046; doi:10.1039/d5sc04173b)
Supplement: SC-016-D5SC04173B-s001 [file SC-016-D5SC04173B-s001.pdf]

Supplementary Information (SI) for Chemical Science.
This journal is © The Royal Society of Chemistry 2025

Supplementary Information for

Towards Large-scale Chemical Reaction Image Parsing via a Multimodal Large Language Model

Yufan Chen, Ching Ting Leung, Jianwei Sun, Yong Huang,
Linyan Li, Hao Chen, and Hanyu Gao*

Contents

1 Materials and Methods **3**
1.1 Synthetic Dataset Generation . . . 3
1.2 Data Augmentation . . . 5
1.3 Language-based Task Instruction for Reaction Image Parsing . . . 5
1.3.1 Reaction Component Identification Task . . . 6
1.3.2 Reaction Condition Interpretation task . . . 7
1.4 Model Architecture . . . 7
1.4.1 Multimodal Encoder . . . 7
1.4.2 Task-Guided ReactionImg Tokenizer . . . 8
1.4.3 LLM Task Decoder . . . 8
1.5 Workflow of Chemical Reaction Image Parsing Using RxnIM . . . 9
1.6 Evaluation Metrics . . . 9
1.6.1 Reaction Component Identification Task . . . 9
1.6.2 Reaction Condition Interpretation task . . . 10
1.7 Implementation Details . . . 11
1.8 RxnIM.web: Interactive Human–AI Collaboration Platform . . . 11

2 Additional Notes **12**
2.1 Statistics of Different Datasets for Image-based Reaction Parsing Tasks 12
2.2 Statistics of Different Datasets on the Reaction Component Identification Task . . . 12
2.3 DOI numbers for Real Reaction Image Examples . . . 13
2.4 Training Settings and Loss . . . 13

3 Additional Discussion **14**
3.1 Detailed Model Performance on the Reaction Component Identification Task on the Synthetic Test Dataset . . . 14
3.2 Error Analysis . . . 15
3.3 Influence of Different Base LLMs . . . 16
3.4 Influence of Input Image Resolution . . . 16

4 Supplementary Figures **20**

1 Materials and Methods

1.1 Synthetic Dataset Generation

To train the reliable RxnIM, we developed an algorithm to artificially create synthetic images similar to real-world reaction images automatically. The algorithm used structured reaction data to construct reaction images following general conventions in chemistry. We used the Pistachio dataset, which includes structured information of reactant and product SMILES, along with reaction conditions such as agents, solvents, temperature, time, and yield. Then we define the reaction patterns and layout rules in single-line, multiple-line, branch, and cycle images. A sub-image generator with OpenCV and Indigo toolkit then creates several sub-images with reaction components like molecular structures, reaction arrows, and condition texts, adhering to common layout rules in chemistry. Since we have complete control of the image generation process, we can automatically record the ground truth for the prediction tasks (e.g. locations of the objects and their roles). These sub-images and ground truth data are synthesized into labeled reaction images by the reaction images generator, which arranges them according to the predefined patterns and layout rules. We show a detailed pipeline of our algorithm in Fig. S1.

We first introduce how to acquire and preprocess individual reaction components (molecular images, reaction conditions, arrows, labels, etc.) to generate synthetic images for training the model. The raw data used to generate the synthetic images comes from Pistachio, a dataset containing millions of chemical reactions. Each reaction data contains detailed information about a single chemical reaction, including SMILES of reactants and products, as well as reaction conditions such as agents, solvents, temperature, and time. Our algorithm first uses the Indigo library to generate molecular images of reactants and products. The generation process generates images of different styles by randomizing the option settings to enhance the diversity of the data. It then extracts information of the reaction condition. For agents and solvents, we chose in a certain proportion whether to use SMILES information to generate molecular images or text information to generate text to further enhance the diversity of data, all other conditions are represented by text. We use the OpenCV library to generate text. The generation process randomizes option settings to generate the text with different font sizes and styles. Chemical labels are created in the same way. Arrows are created by the OpenCV library and are randomly styled and sized. In the process of generating each element, we record the size of the element so that the corresponding coordinates can be directly obtained in the final generated reaction image to generate the ground truth of the data automatically.

Then, we describe the process of synthesizing single-line and single-step reaction images using the components generated in the previous section and then expand to more complex-style reaction images. Synthetic single-line and single-step reaction images are built on a blank canvas, where individual elements such as molecular images, reaction conditions, arrows, and labels are placed according to pre-defined patterns. Our algorithm specifies the absolute and relative positions of each component to ensure that the generated images resemble real chemical reaction maps. The synthesis process starts with initializing a blank canvas, usually set to a fixed size

to accommodate various reaction schemes. The positions of reactants and products on the canvas are determined by their sizes, which are recorded during the generation phase. Reactants are placed on the left side of the canvas, while products are placed on the right. A generated reaction arrow is added between them, indicating the direction of the reaction, and the reactants are connected with randomly styled " + " symbols. A randomly generated series of numbers is placed below each molecule image to represent the chemical label. Agents and solvents are represented as either molecular images or text, depending on randomized component generation. All other conditions are represented by text. These conditions are regularly placed above or below the reaction arrow to clearly illustrate the reaction process. The precise position of each component on the canvas is carefully calculated to prevent overlap and to ensure a clear and coherent reaction scheme. The algorithm dynamically adjusts the component positions according to the size and number of components. This flexibility allows the generation of diverse single-step reaction images that can handle different amounts of reactants, products, and conditions without compromising the visual clarity of the image. Once all the components are placed, the algorithm merges the image by blending all the elements onto the canvas. The resulting image is a comprehensive reaction map, including all necessary molecular images, arrows, labels, and condition annotation. In addition, the algorithm records the coordinates and the categories of each component, the characters and the condition roles in each condition text region in the image. This information is used to generate ground truth data.

Reaction images with more complex patterns, such as multiple-line, branch, and cycle, are then generated by modifying and merging single-line and single-step reaction images according to the pre-defined layouts of the reaction patterns. We synthesize complex reaction patterns in two stages, as shown in the Fig. S1. Stage one builds single-line multi-step sub-images from many primitive single-line single-step images. For diversity, we also create an inverse-direction variant of each step. To assemble a multi-step sub-image, we keep the first step intact and, for every later step, crop away the left reactant region while keeping the arrow, product, and conditions. The cropped steps are then concatenated one by one to the right of the growing chain. Although any length is possible, we cap the number of steps at four to stay realistic.

With these sub-images in hand, we compose multiple-line reaction images in two ways. In the first, where the page shows several independent reactions, we vertically stack multiple single-line multi-step sub-images and, if needed, pad the canvas to a square and optionally insert small reagent/condition boxes near selected steps. In the second, a single reaction wraps across rows. We split a single-line multi-step sub-image into several rows and add a continuation arrow at the right end of each row except the last; optionally we add a counterpart at the left of rows except the first to indicate wrap-back. Reaction conditions are placed above or below these arrows. The rightmost molecule of a row is treated as the reactant for the leftmost molecule of the next row so the step-to-step flow is preserved.

For branch patterns, we start from a single-line multi-step sub-image, choose a branching step, and insert vertical arrows with corresponding conditions between rows. This indicates that the pathway can continue horizontally along the original row or descend to an alternative route starting from the same step.

For cycle patterns, we take a single-line multi-step sub-image and generate a reversed-direction counterpart with the same number of steps, placing components from right to left. We concatenate the two sub-images vertically and add a downward arrow on the far right and an upward arrow on the far left to close the loop with corresponding conditions. In addition, our algorithm will adjust the relative positions of different reaction steps so that the synthetic image resembles the real circular cycle reaction image.

Throughout, the model is trained to learn the flow and layout of reactions rather than their chemical meaning, so modifying and merging steps drawn from different reactions is acceptable and often necessary for generating realistic composites.

Finally, we utilized the algorithm to generate 60,200 synthetic images and the corresponding ground truth, where each reaction entry is unique, ensuring that no duplicate reactions were used during image generation. This effectively solved the problem of the lack of large-scale diversified chemical reaction image datasets and provided strong support for training the model. More detailed dataset statistics, including the number of images of each type and the number of reaction entries per image, can be found in SI Sections 2.1 and 2.2.

1.2 Data Augmentation

To further increase dataset diversity and improve the robustness of RxnIM, we developed a two-stage data augmentation strategy. In the first stage, compositional augmentation was applied to synthesize more complex reaction diagrams. Specifically, multiple reaction images were randomly sampled from the training set and concatenated vertically to form a new multi-reaction diagram. When the sampled diagrams had different widths, a random horizontal offset was introduced to align them. The number of diagrams concatenated together ranged from two to five, with the probability of sampling larger compositions decreasing exponentially. The annotations of the original diagrams were merged, and entity bounding boxes were shifted accordingly to match the new spatial layout.

In the second stage, we applied image-level transformations to further expand the dataset and simulate realistic image perturbations. These included resizing, padding, rotation, horizontal flipping, Gaussian noise injection, and color jittering, each applied at random. Together, the compositional augmentation and image-level transformations produced a richer training corpus, enabling RxnIM to better generalize across diverse reaction layouts and imaging conditions.

1.3 Language-based Task Instruction for Reaction Image Parsing

We introduce the language-based task instructions for the reaction component identification task, and the reaction condition interpretation task. This design can also be further used to customize more reaction component identification task instructions. We describe the tasks by providing a task description with placeholders and special reaction role tokens and specifying the desired output format via task instructions.

1.3.1 Reaction Component Identification Task

We construct the reaction component identification task as an extension of object detection. Previous works [1–3] represent each object as five tokens i.e., Object = $[x_{min}, y_{min}, x_{max}, y_{max}, class]$, where first four tokens describe its bounding box in the image, (x_{min}, y_{min}) and (x_{max}, y_{max}) are the coordinates of the top-left and bottom-right points, respectively. They also quantize the continuous image coordinates into extra discrete tokens by binning ($e.g., < bin_0 >, \ldots, < bin_{999} >$). We made a few modifications to represent the object more clearly and accurately. First, we represent coordinates using numbers directly (*e.g.*, use three single-digit original number tokens to represent a coordinate from 0 to 999). We further discuss the different performance between these two representations in the Effect of Model Components and Configurations section. Second, we allocate a unique ID token following the class token for each object, enabling the model to represent each object more accurately. Then the class token represents the object type i.e. $class =$ [Str] or [Txt]. We define two classes of objects: molecule structure ([Str]) and text description ([Txt]). Usually, reactants and products are represented as molecular structures, and conditions are described in text in the image. Finally, each object in the reaction image is represented as $\text{Object}_i = [x_{min}, y_{min}, x_{max}, y_{max}, class, \text{ID}_i]$. This format is also used as the output in the conventional object detection task during the first stage of our training. We then extend this format into the reaction component identification output by following a specialized reaction sequence:

$$
\begin{aligned}
&\text{Reaction} = \textbf{[Rxn/st]} \quad \text{Reactant} \quad \text{Condition} \quad \text{Product} \quad \textbf{[Rxn/ed]},\\
&\text{Reactant} = \textbf{[Rct/st]} \quad \text{Object}_i * n \quad \textbf{[Rct/ed]},\ (n > 0)\\
&\text{Condition} = \textbf{[Cnd/st]} \quad \text{Object}_i * m \quad \textbf{[Cnd/ed]},\ (m \geq 0)\\
&\text{Product} = \textbf{[Prd/st]} \quad \text{Object}_i * n \quad \textbf{[Prd/ed]},\ (n > 0)
\end{aligned}
$$

Where each reaction is a sequence starting with a [Rxn/st] token, which consists of three reaction roles: Reactant, Condition, and Product, and ends with a [Rxn/ed] token. Each reaction role is a subsequence of objects that begins with a start token ([Rct/st],[Cnd/st] or [Prd/st]), and ends with an end token ([Rct/ed],[Cnd/ed] or [Prd/ed]). The conditions $n > 0$ and $m \geq 0$ indicate that the Reactant and Product must contain at least one object. However, the Condition can be empty. Through the language-based task instruction, we ask the model to search all the reactions existing in the reaction image from the top left corner of the image according to this special reaction sequence, and finally output a set of reaction sequences. An example of language task instruction for the reaction component identification is as follows:

Please list every reaction in this image[image] in detail. For each reaction, include the category and unique ID of each object, along with their coordinates [x1, y1, x2, y2]. Categories include Structure ([Str]) and Text ([Txt]). Describe their roles in each reaction ([Rxn/st] to [Rxn/ed]), including Reactants ([Rct/st] to [Rct/ed]), Conditions ([Cnd/st] to [Cnd/ed]), and Products ([Prd/st] to [Prd/ed]). Note that Reactants and Products must include at least one object, while Conditions can be specified without any objects. Structured output format should

be: [Rxn/st][Rct/st](object 1)$\cdots$ [Rct/ed][Cnd/st](object 2)$\cdots$ [Cnd/ed][Prd/st](object 3)$\cdots$ [Prd/ed][Rxn/ed],[Rxn/st]$\cdots$. Only the Conditions section can be empty (i.e., [Cnd/st][Cnd/ed] without anything between).

Where [image] is the Placeholder for the image token. The image tokens will replace it during training.

1.3.2 Reaction Condition Interpretation task

The reaction condition interpretation task is designed as an extension of scene text-centric visual question answering. For this task, our model only focuses on the regions that are recognized as the text description ([Txt]) of the condition in the image by the previous reaction component identification task. We define the reaction condition interpretation output format as follows:

$$\text{Condition Role} = [\text{"Text"} \ \ [Role]] * n, \ \ (n > 0)$$

Where "Text" represented the text recognition results of a single word and followed by a special condition role token to indicate its role in condition, i.e. $Role = [\text{Agt}], [\text{Svt}], [\text{Tem}], [\text{Time}], \text{or} \ [\text{Yld}]$. We define five classes of conditional roles that frequently occur in reactions: Agent([Agt]), Solvent([Svt]), Temperature([Tem]), Time([Time]), and Yield([Yld]). We ask the model to identify all discrete words from a condition text region in the image and assign a condition role token to each word by task instruction. Any extracted text whose role is not in the five labels, the model will skip the role token. We describe the language task instruction for the reaction condition interpretation as follows:

For the given image[image], what words are written in this text box[objs]. And please indicate the condition role[Role] of each word in: solvent[Svt], agent[Agt], temperature[Agt], time [Time] and yield[Yld]. Note that any extracted text whose role is not in the five labels, skip the [Role] token. Structured output format should be:'Text content'[Role],$\cdots$.

Where [objs] is the Placeholder for the corresponding text region. It will be replaced by the bounding box of the text region during training.

1.4 Model Architecture

1.4.1 Multimodal Encoder

In the core model architecture (Fig. 1(b)), we propose a multimodal encoder that jointly processes chemical reaction images and task-specific textual instructions to produce multi-scale, task-aware feature maps. The encoder consists of an image encoder and a text encoder, whose outputs are aligned through cross-attention.

Specifically, given a reaction image of height H and width W, we feed it into a ResNet [4] backbone composed of four sequential stages. Each stage produces a feature map F_c^v with progressively reduced spatial resolution: $H/4 \times W/4$, $H/8 \times W/8$, $H/16 \times W/16$, and $H/32 \times W/32$. These feature maps capture multi-scale representations of molecular structures, arrows, and textual labels present in the reaction diagram.

Meanwhile, the task instruction is processed by a BERT encoder [5], which outputs a sequence of language feature tokens F_c^l. To integrate the modalities, each ResNet feature map is first reshaped into a sequence of visual tokens while preserving its spatial grid. These visual tokens are projected into a shared embedding space and then fused with the language tokens using a standard cross-attention mechanism [6]. The attention score between a visual token q_v and a textual token k_l is computed as the scaled dot product:

$$\text{Attention}(q_v, k_l) = \text{softmax}\left(\frac{q_v W_Q \cdot (k_l W_K)^\top}{\sqrt{d_k}}\right)$$

where W_Q and W_K are learned projection matrices and d_k is the dimension of the key vectors. This procedure is performed independently for each scale, resulting in a set of multi-scale task-aware feature maps $F_c^{v,l}$ that encode both fine-grained visual information and high-level language guidance. By explicitly conditioning visual features on textual instructions at multiple resolutions, the multimodal encoder aligns molecules, arrows, and condition texts with their semantic roles, enabling robust recognition and grounding across various reaction images.

1.4.2 Task-Guided ReactionImg Tokenizer

Unlike previous approaches [7–9] that represent images using fixed-size patches directly fed into the LLM decoder, we propose a task-guided ReactionImg tokenizer that flexibly generates image tokens aligned with task-specific instructions.

Our tokenizer is built upon a transformer-based encoder–decoder architecture, deformable DETR [10], to capture high-level image representations. In the encoder, a multi-scale deformable cross-attention module [10] aggregates information across the task-aware multi-scale feature maps obtained from the multimodal encoder. The encoder outputs retain the same spatial resolutions as the inputs, with both keys and queries defined as pixels from the multi-scale feature maps.

During decoding, we use the new feature maps as keys and initialize N learnable queries $Q = \{q_i\}_{i=1}^N$ to extract N image tokens $T = \{(e_i, p_i)\}_{i=1}^N$. Each image token consists of an embedding vector e_i, encoding semantic information, and a position vector p_i, encoding spatial coordinates. This design enables the tokenizer to represent not only individual objects in reaction images but also their spatial relationships.

Furthermore, by conditioning the extracted tokens on task-specific instructions, the tokenizer produces visual representations that are more informative and targeted to the downstream tasks. This ensures that the model learns visual features that are both semantically meaningful and directly useful for chemical information extraction.

1.4.3 LLM Task Decoder

Our task decoder is built based on a widely used LLM, Llama [11], and is designed to handle various chemical reaction image parsing tasks with task instructions. However, Llama lacks the specialized tokens needed for these chemical reaction

image parsing tasks. Therefore, we expand the vocabulary of Llama with special tokens designed for reaction parsing tasks as mentioned in Sec. 1.3, including object tokens([Str], [Txt]), reaction token(([Rxn/st], [Rxn/ed]), reaction role tokens([Rct/st], [Rct/ed], [Cnd/st], [Cnd/ed], [Prd/st], [Prd/ed]), and condition role tokens([Agt], [Svt], [Tem], [Time], [Yld]). Furthermore, following [3], we apply the output-format-as-query decoding strategy to address the inefficiency of the causal LLM model. The LLM model first parses the task instructions into structured output format(*e.g.*, "[Rxn/st] [Rct/st] [$x_{min}, y_{min}, x_{max}, y_{max}, class, \mathrm{ID}_i$] ... [Rct/ed] ... [Rxn/ed]" for reaction component identification task, "'Text'[*Role*]" for reaction condition interpretation task). The tokens in the structured output format are then provided as queries to the decoder to generate the desired output based on the query. This approach enables the model to avoid inefficient token-by-token decoding when parsing chemical reaction images, particularly in tasks involving visual perception and positioning. It strictly constrains the output to the specified structure, such as a reaction schema or condition role list, while maintaining a unified framework for these tasks. Through this method, we convert these tasks into the token classification format, so that the cross-entropy loss can be applied to train and fine-tune the model.

1.5 Workflow of Chemical Reaction Image Parsing Using RxnIM

After training the RxnIM model, we designed a workflow to obtain the final reaction image parsing results as shown in Fig. 1(c). The model first outputs the result of the reaction component identification task for the entire image. Blue, red, and orange boxes represent reactants, conditions, and products in reaction roles, respectively. The categories of the objects are indicated in green boxes. Following this, it focuses on the regions identified as text description in condition, applying OCR and role identification to extract the information of condition roles.

The results from both tasks are integrated, providing comprehensive data that combines structural and descriptive details of the reaction. Additionally, a molecular structure recognition model post-processes these results, converting visual molecular structures into machine-readable data formats like SMILES or Molfile. This conversion is crucial for the practical use of the data in synthetic chemistry applications. The final output is structured data of the chemical reaction as shown in the bottom of Fig. 1(c), ready for documentation and computational analysis.

1.6 Evaluation Metrics

1.6.1 Reaction Component Identification Task

Evaluating the results for the reaction component identification task is complex due to the nature of the predictions and the ground truth, which are sets of reaction structures. In many cases, the predictions may not align perfectly with the ground truth. For instance, the bounding boxes for entities might be slightly misaligned, or the predicted order of reactions might differ. Despite these discrepancies, many such instances should still be deemed correct. Following [1], We use two groups of evaluation metrics, hard match and soft match, to evaluate the model.

Specifically, We start by comparing a single predicted reaction, denoted as $\hat{R}$, with a single ground truth reaction, R. This involves establishing a mapping between the lists of objects in $\hat{R}$ and R. For each object in $\hat{R}$, we identify the corresponding entity in $\hat{R}$ that has the highest bounding box overlap. This overlap is quantified by the intersection over union (IoU) score. For consistency with the previous benchmark [1], if the maximum IoU exceeds a threshold of 0.5, the predicted and ground truth bounding boxes are considered to have successfully matched.

Furthermore, a prediction $\hat{R}$ is considered a match with the ground truth R in the hard match evaluation only if all reactants, conditions, and products between $\hat{R}$ and R can be aligned perfectly. In contrast, the soft match evaluation only focuses on molecule objects and does not differentiate between reactants and agents, which are part of the conditions. The rationale for using soft match evaluation is twofold. First, it considers only molecular objects and not textual objects, addressing the common question of how consecutive lines of text are annotated, e.g., viewed as a single object or multiple objects. Second, it avoids distinguishing between reactants and conditions. This is because, in certain cases, a molecule may appear above or below the reaction arrow, which is mainly used for placing reaction conditions, but functionally acts as a reactant due to its significant contribution of heavy atoms. This ambiguity about the separation between reactants and agents is tolerable in chemistry.

In each reaction image, the ground truth is represented as $G_r = \{R_1, R_2, \ldots, R_n\}$, and the prediction is represented as $P_r = \{\hat{R}_1, \hat{R}_2, \ldots, \hat{R}_m\}$. Then we compute the precision, recall, and F_1 scores for hard match and soft match, respectively. Since we do not have a one-to-one correspondence between the predicted reactions and the ground truth reactions, we list all pairs and compare each $\hat{R}_i$ to each R_j. The metric is defined as follows:

$$\text{Precision} = \frac{1}{m} \sum_{j=1}^{m} \mathbb{I}(\exists i \in \{1, \ldots, n\}, \hat{R}_j \text{ matches } R_i) \tag{1}$$

$$\text{Recall} = \frac{1}{n} \sum_{i=1}^{n} \mathbb{I}(\exists j \in \{1, \ldots, m\}, R_i \text{ matches } \hat{R}_j) \tag{2}$$

$$F_1 = \frac{2 \cdot \text{Precision} \cdot \text{Recall}}{\text{Precision} + \text{Recall}} \tag{3}$$

1.6.2 Reaction Condition Interpretation task

For the reaction condition interpretation task, we use OCR accuracy to determine the proportion of correctly identified characters across all characters. For the role identification, only words with an OCR accuracy above a threshold of 0.8 are included in the calculations. The condition role identification (CRI) accuracy is then used to determine the proportion of words correctly classified by the model. Additionally, the conventional precision, recall and F_1 are utilized to analyze the performance of the model in each specific condition role. The OCR accuracy and CRI accuracy is defined as follows:

$$\text{OCR accuracy} = \frac{\text{Number of Correct Characters}}{\text{Total Number of Characters}} \tag{4}$$

$$\text{CRI accuracy} = \frac{\text{Number of Correct Predictions}}{\text{Total Number of Predictions}} \tag{5}$$

1.7 Implementation Details

In our RxnIM, we employ ResNet-50 [4] as the image encoder and BERT-Base [5] as the text encoder within our multimodal encoder framework. For the task-guided ReactionImg tokenizer, we adopt deformable DETR (D-DETR) [10] to capture high-level information and extract image tokens. We set the number of queries and image tokens M to 300. And the number of encoder/decoder layers is set to 6 for D-DETR. Subsequently, we integrate Llama-2-7B [12], an advanced version of the Llama [11] model, as our LLM-based task decoder to handle different tasks effectively.

The training of the model is structured in three stages. In the first stage, We start by loading pre-trained weights for D-DETR, BERT, and Llama-2-7B. During this phase, we train the multimodal encoder and the ReactionImg tokenizer, while the LLM's parameters remain frozen. The focus is on the conventional object detection task to enable the model to first locate objects in reaction images accurately. In the second stage, all model parameters are fine-tuned. The training, which lasts for 30 epochs, involves the reaction component identification task and the reaction condition interpretation task using the synthetic dataset split in a 7:3 ratio. The initial learning rate is set at 2×10^{-4}. In the final stage, we only fine-tune the LLM while freezing all other parameters. The training utilizes the real-image dataset for the reaction component identification task, adjusting the data split to 8:2, focusing on parsing complex reaction image patterns over 50 epochs with an initial learning rate of 2×10^{-5}.

We employ AdamW as the optimizer with a cosine annealing learning rate schedule throughout all training stages. The model is trained using 8 × NVIDIA H800 GPUs, ensuring robust computational support for our extensive training regimen. The complete training settings and training loss can be found in the SI Section 2.4.

1.8 RxnIM.web: Interactive Human–AI Collaboration Platform

The RxnIM.web application has been developed using Gradio, a Python-based web framework, and is hosted on Hugging Face Spaces. This lightweight and flexible setup enables easy deployment through an intuitive web interface.

Upon launching, the web app initializes a server to handle user requests and preloads our RxnIM model for reaction image parsing as well as a molecular recognition model for converting molecular objects into SMILES strings. Hosting these models on Hugging Face ensures efficient scaling and seamless updates without significant downtime. When a user uploads a reaction image file, the image is processed following the previously described workflow. The outputs are aggregated and displayed in the web interface, allowing users to visualize parsed editable reaction data in both human-readable and machine-readable formats.

To facilitate reliable dataset construction, we also proposed a Human-AI collaboration workflow based on RxnIM.web. Specifically, since the RxnIM.web supports editable and visualized ChemDraw-style reaction outputs. Chemists can simply

compare the original reaction image with the output, quickly identify potential misclassifications, and directly edit molecules or conditions within the same interface, which is similar to working in ChemDraw. This streamlined correction process efficiently reduces predictive noise when building high-quality reaction datasets, while maintaining an intuitive user experience.

For efficient performance, the Gradio-based interface enables real-time interaction, and the backend leverages the computational resources of Hugging Face Spaces to manage model inference tasks in parallel with minimal latency. RxnIM.web is openly accessible at https://huggingface.co/spaces/CYF200127/RxnIM.

2 Additional Notes

2.1 Statistics of Different Datasets for Image-based Reaction Parsing Tasks

We offer a comprehensive analysis of the synthetic and real datasets in Supplementary Table S1, detailing the number of samples allocated for training, validation, and testing in two main tasks: the reaction component identification task and the reaction condition interpretation task. While the synthetic dataset includes data for the conventional object detection task, it is not presented here, as it is not part of the reaction parsing tasks.

Table S1: **Statistics of different datasets for image-based reaction parsing tasks.** We provide a detailed breakdown of the synthetic and real datasets, including the number of samples used for training, validation, and testing across the reaction component identification task and reaction condition interpretation task.

Dataset	Task	Train Sample	Valid Sample	Test Sample
Synthetic	Reaction Component Identification	48160	6020	6020
	Reaction Condition Interpretation	166116	20765	20764
Real	Reaction Component Identification	1240	138	138
	Reaction Condition Interpretation	–	–	–

2.2 Statistics of Different Datasets on the Reaction Component Identification Task

We show the amount and the complexity of different reaction image patterns based on the average number of reactions and objects per image in Supplementary Table S2. For the synthetic dataset, Single-Line images average 1.12 reactions and 7.68 objects per image, while Multiple-Line images have 3.35 reactions and 13.87 objects per image. Branch images show an increased complexity with 4.35 reactions and 14.54 objects per image. Cycle images are the most complex, averaging 6.11 reactions and 15.74 objects per image. In the real dataset, Single-Line images average 1.21 reactions and 10.32 objects per image, Multiple-Line images have 3.65 reactions and 18.58 objects

Table S2: **Statistics of different datasets on the reaction component identification task on four different patterns of reaction images.** We summarize the data distribution across synthetic and real datasets for four distinct reaction image patterns: Single-Line, Multiple-Line, Branch, and Cycle. The table includes the count of images, objects, and reactions, and the average numbers of objects and reactions per image, illustrating the varying complexities of the four reaction image patterns and the comprehensive scope of the dataset used for training and evaluating the RxnIM.

Dataset	Count Type	Single-Line	Multiple-Line	Branch	Cycle
Synthetic	images	18060	18060	12040	12040
	objects	138702	250649	174995	189564
	reactions	20163	60421	52380	73622
	avg. objects per image	7.68	13.87	14.54	15.74
	avg. reactions per image	1.12	3.35	4.35	6.11
Real	images	730	260	286	102
	objects	7536	4831	4934	1926
	reactions	882	948	1313	633
	avg. objects per image	10.32	18.58	17.25	18.88
	avg. reactions per image	1.21	3.65	4.59	6.21

per image, Branch images show 4.59 reactions and 17.25 objects per image, and Cycle images exhibit the highest complexity with 6.21 reactions and 18.88 objects per image. The table reveals the increasing complexity from Single-Line to Cycle images, reflecting the diversity and challenge posed by different reaction patterns. This diversity is crucial for training and evaluating models, ensuring comprehensive coverage of various chemical reaction scenarios.

2.3 DOI numbers for Real Reaction Image Examples

The DOI numbers of the relevant journal articles corresponding to the real reaction image examples used in the main text are provided in Supplementary Table S3. The examples include various figures and predictions demonstrated in the main text and Supplementary Information. The DOIs are essential for tracing the specific reactions and understanding their context within the broader chemical literature.

2.4 Training Settings and Loss

We summarize the complete training settings of our three-stage training in Supplementary Table S4 to facilitate reproducibility. The table provides a detailed summary of the datasets, frozen components, and tasks involved in the three stages of training. It also outlines the hyperparameters used to fine-tune the model progressively from synthetic datasets in the initial stages to real-world data in the final stage. Each stage is tailored to specific tasks and components to ensure efficient learning and optimal model performance.

Table S3: DOI numbers of the relevant journal articles for Real Reaction Image Examples. We provide the DOI numbers of the relevant journal articles corresponding to the real reaction image examples used in the main text. The table lists each reaction image example and its respective available DOI number, enabling readers to access the original sources.

Reaction Image Example	DOI Numbers
The example in Fig. 1(b)	acs.oprd.5b00371
Fig. 3, Prediction 1	acs.orglett.5b01044
Fig. 3, Prediction 2	ol052245s
Fig. 3, Prediction 3	jo982004
Fig. S2	acs.orglett.5b01872
Fig. S3	acs.joc.5b00632
Fig. S4	acs.orglett.6b01181
Fig. S5	jo2001534
Fig. S6	ol036510q
Fig. S7	op8002486
Fig. S8, Prediction 1	op8002486
Fig. S8, Prediction 2	op8002486
The example in Fig. S9	acs.oprd.5b00209

To further illustrate the effectiveness of different training strategies, we visualize the training loss curves under five settings: Stage 1 only, Stage 1 + 2, Stage 1 + 3, Stage 2 + 3, and All Stages. As shown in Fig. S10, all strategies exhibit consistent downward trends. The Stage 1 segments are identical across all strategies that include it. Among them, the full three-stage pipeline (All Stages) achieves the lowest final loss, demonstrating the benefit of progressive fine-tuning. Other strategies, such as Stage 1 + 3 and Stage 2 + 3, converge to slightly higher loss values, indicating that omitting any stage results in suboptimal learning. This comparison further validates the importance of each stage in our training strategy.

3 Additional Discussion

3.1 Detailed Model Performance on the Reaction Component Identification Task on the Synthetic Test Dataset

We compare the performance of the four best models on the Reaction Component Identification Task on four different patterns of reaction images on the synthetic test dataset in Supplementary Table S5, reinforcing the findings from the real test dataset. RxnIM consistently outperforms the other models across all evaluation metrics and reaction image patterns. Notably, the performance gap between RxnIM and the second-best model, RxnScribe, is even more pronounced in the synthetic dataset. For instance, in the hard match criteria, RxnIM achieves an F_1 score of 81.2% in branch

Table S4: **Training configuration settings for different stages.** The table summarizes the datasets, frozen components, tasks, and hyperparameters used during the three stages of training.

**Settings**	**Stage 1**	**Stage 2**	**Stage 3**
Real Dataset	✗	✗	✓
Synthetic Dataset	✓	✓	✓
Freeze LLM	True	False	False
Freeze BERT	False	False	True
Freeze D-DETR	False	False	True
Object Detection Task	✓	✗	✗
Reaction Component Identification Task	✗	✓	✓
Reaction Condition Interpretation Task	✗	✓	✓
Epochs	20	30	50
Learning Rate	2e−4	2e−4	2e−5
Learning Rate Schedule	cosine decay	cosine decay	cosine decay
Optimizer	AdamW	AdamW	AdamW
Optimizer Hyper-parameters	$\beta_1, \beta_2 = 0.9, 0.999$	$\beta_1, \beta_2 = 0.9, 0.999$	$\beta_1, \beta_2 = 0.95, 0.999$
Weight Decay	1e−5	0.00	0.00
Input Size	1333×1333	1333×1333	1333×1333
Drop Rate	0.0	0.0	0.0
GPUs for Training	$8 \times$ H800	$8 \times$ H800	$8 \times$ H800

images, compared to RxnScribe's 69.2%, GPT-o3's 54.6%, and ReactionDataExtractor 2.0's 46.2%. Similarly, in cycle images, RxnIM attains an F_1 score of 72.8%, while RxnScribe reaches only 59.6%, GPT-o3 reaches only 27.0%, and ReactionDataExtractor 2.0 reaches only 27.3%. These results indicate that RxnIM's robust image reasoning and localization capabilities are further highlighted in the controlled conditions of synthetic data. The consistent superiority across both real and synthetic datasets not only validates the model's effectiveness but also underscores its generalizability and reliability in handling a wide range of reaction image complexities.

3.2 Error Analysis

We conducted a detailed error analysis of RxnIM predictions on the synthetic dataset to understand the performance of the model and identify directions for further improvement. As shown in Fig. S11, molecular recognition errors represent the largest proportion of mistakes, accounting for approximately 72% of the total errors. These errors mainly involve incorrect or incomplete generation of molecular SMILES from the visual representations. Missing reaction components constitute about 15% of errors, primarily involving small or partially occluded reactants and products. Condition interpretation errors, in which condition texts were misclassified or incorrectly extracted, accounted for 10% of the total errors. Finally, instances where the entire reaction was missed were the least common, making up only 3% of the errors. These results indicate that future work should prioritize improving molecular recognition

accuracy, potentially through integrating a stronger molecular recognition model, as this would yield the largest overall improvement.

3.3 Influence of Different Base LLMs

We show the different base LLMs [11, 12] result in varying model performance on the reaction component identification task and the reaction condition interpretation task in Supplementary Table S6. Llama-7B and Llama-13B models, while generally competent, showed slightly lower performance in both precision and recall compared to the newer Llama-2 models, particularly under the hard match criteria. Llama-2-13B exhibited higher scores across most metrics, reflecting its ability to handle complex reaction image parsing and recognition tasks more effectively. However, it also required significantly longer training times per image, indicating a higher computational cost. The Llama-2-7B model, on the other hand, offered a favorable balance between performance and efficiency. It achieved nearly the highest precision, recall, and F_1 scores across both synthetic and real datasets on the reaction component identification task, as well as OCR and CRI accuracy on the reaction condition interpretation task. The model's training time per image was also notably shorter than Llama-2-13B, making it more practical for large-scale or resource-constrained environments. Thus, Llama-2-7B was selected as the preferred base LLM for the RxnIM framework, providing an optimal balance of accuracy, efficiency, and computational resource utilization.

3.4 Influence of Input Image Resolution

We display the effect of using different input image resolutions for our RxnIM on the two tasks in Supplementary Table S7. The table reveals a trend where increasing image resolution generally enhances model performance across all evaluated metrics. Higher resolutions provide more detailed visual information, which improves the model's ability to accurately identify and classify components in reaction images. For synthetic datasets, the highest performance in terms of hard match F_1 score, soft match F_1 score, OCR accuracy, and CRI accuracy was achieved at the 1333×1333 resolution. Similarly, for real datasets, the same resolution provided the best precision, recall, and F_1 scores under hard and soft match criteria.

Despite the slight increase in training time per image as resolution increases, the significant improvement in performance metrics justifies the choice. The 1333×1333 resolution offers the most detailed and accurate representation of reaction images, making it the optimal choice for our RxnIM framework. This resolution provides a balance between computational efficiency and the need for high-quality image data, ensuring the model's effectiveness in diverse and complex chemical image scenarios.

Table S5: **Comparison of model performance on the reaction component identification task on four different patterns of reaction images on synthetic test dataset (scores are in %)**. We also provide a detailed comparison of model performances across different evaluation metrics for synthetic datasets, encompassing four reaction image patterns. The table highlights the precision, recall, and F_1 scores under both hard and soft match criteria, showcasing the effectiveness of our model in accurately extracting reaction data from synthetic images.

Evaluation Metric	Model	Single-Line			Multiple-Line			Branch			Cycle		
		Precision	Recall	F_1	Precision	Recall	F_1	Precision	Recall	F_1	Precision	Recall	F_1
Hard Match	GPT-o3 [13]	78.4	70.4	74.1	71.2	60.9	65.6	59.5	50.6	54.6	32.5	23.5	27.0
	ReactionDataExtractor 2.0 [14]	78.0	72.1	75.0	69.5	63.6	66.3	50.1	42.8	46.2	30.4	24.7	27.3
	RxnScribe [1]	87.7	86.4	87.0	81.6	77.9	79.7	73.2	65.8	69.2	62.7	56.8	59.6
	RxnIM	**91.2**	**90.5**	**90.8**	**85.3**	**84.1**	**84.7**	**83.7**	**78.9**	**81.2**	**75.2**	**70.5**	**72.8**
Soft Match	GPT-o3 [13]	83.5	77.5	80.3	74.6	67.9	71.1	65.4	58.3	61.5	34.6	27.2	30.4
	ReactionDataExtractor 2.0 [14]	87.1	82.0	84.5	79.0	73.9	76.3	59.3	53.2	56.1	46.0	37.9	41.5
	RxnScribe [1]	92.7	92.2	92.4	90.5	85.4	87.9	84.2	75.5	79.6	77.8	68.8	73.0
	RxnIM	**95.5**	**95.5**	**95.5**	**92.1**	**89.5**	**90.8**	**88.8**	**84.2**	**86.4**	**86.9**	**80.1**	**83.4**

Table S6: **Comparison of Model Performance with Different Base LLMs (Scores are in %).** We summarize the performance of different base LLMs used in our RxnIM on the two tasks. The metrics include training time per image, precision, recall, and F_1 scores under both hard and soft match criteria, as well as OCR accuracy and CRI accuracy. The results are presented for both synthetic and real datasets, providing a comprehensive overview of how different LLMs impact the overall model performance.

Dataset	Base LLM	Training Time per Image ↓	Hard Match			Soft Match			OCR Accuracy	CRI Accuracy
			Precision	Recall	F_1	Precision	Recall	F_1		
Synthetic	Llama-7B	**42 ms**	83.5	82.6	83.0	88.4	88.0	88.2	94.2	93.2
	Llama-13B	81 ms	84.1	82.8	83.4	88.5	87.8	88.1	94.3	93.4
	Llama-2-7B	45 ms	**86.4**	85.9	**86.2**	91.6	90.8	91.2	94.9	**93.6**
	Llama-2-13B	82 ms	86.2	**86.0**	86.1	**91.8**	**91.1**	**91.4**	**95.0**	**93.6**
Real	Llama-7B	**46 ms**	72.6	69.1	70.8	86.1	81.7	83.8	–	–
	Llama-13B	85 ms	72.9	69.1	70.9	87.2	82.0	84.5	–	–
	Llama-2-7B	47 ms	74.7	69.7	72.1	86.9	**82.8**	84.8	–	–
	Llama-2-13B	85 ms	**75.0**	**69.8**	**72.3**	**87.8**	82.5	**85.1**	–	–

Table S7: **Comparison of Model Performance with Different Image Resolutions (Scores are in %).** We present the performance of our RxnIM using various input image resolutions. The results cover both synthetic and real datasets, offering a detailed comparison of how different image resolutions affect the model's effectiveness across the two tasks.

Dataset	Image Resolution	Training Time per Image ↓	Hard Match			Soft Match			OCR Accuracy	CRI Accuracy
			Precision	Recall	F_1	Precision	Recall	F_1		
Synthetic	224 × 224	**16 ms**	73.4	71.5	72.4	77.9	75.8	76.8	84.9	82.7
	448 × 448	<u>24 ms</u>	78.6	77.4	78.0	82.4	81.8	82.1	90.1	89.4
	896 × 896	45 ms	<u>82.4</u>	<u>81.6</u>	<u>82.0</u>	<u>88.1</u>	<u>87.8</u>	<u>87.9</u>	<u>93.4</u>	<u>92.5</u>
	1333 × 1333	63 ms	**86.4**	**85.9**	**86.2**	**91.6**	**90.8**	**91.2**	**94.9**	**93.6**
Real	224 × 224	**17 ms**	59.1	58.3	58.7	65.6	65.9	65.8	–	–
	448 × 448	<u>26 ms</u>	66.4	64.8	65.6	72.4	71.5	71.9	–	–
	896 × 896	47 ms	<u>72.1</u>	<u>66.7</u>	<u>69.3</u>	<u>78.4</u>	<u>78.5</u>	<u>78.5</u>	–	–
	1333 × 1333	66 ms	**74.7**	**69.7**	**72.1**	**86.9**	**82.8**	**84.8**	–	–

4 Supplementary Figures

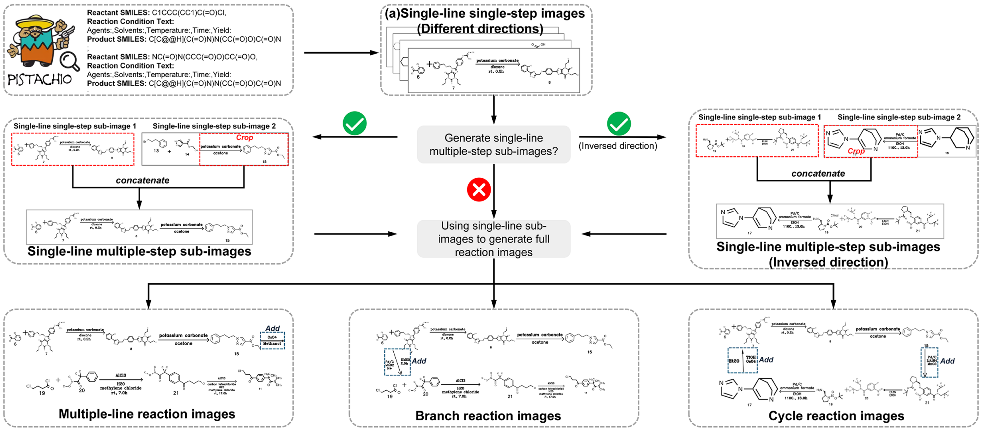

Fig. S1: **Detailed Pipeline of the Synthetic Data Generation Algorithm.** We first render single-line, single-step images (including reversed variants) and build a single-line multi-step sub-image by keeping the first step and cropping the left reactant from later steps, then concatenating up to four steps. These sub-images are arranged with predefined layouts to produce multiple-line, branch, and cycle images, adding arrows and conditions needed; the model learns layout only, so steps from different reactions can be merged.

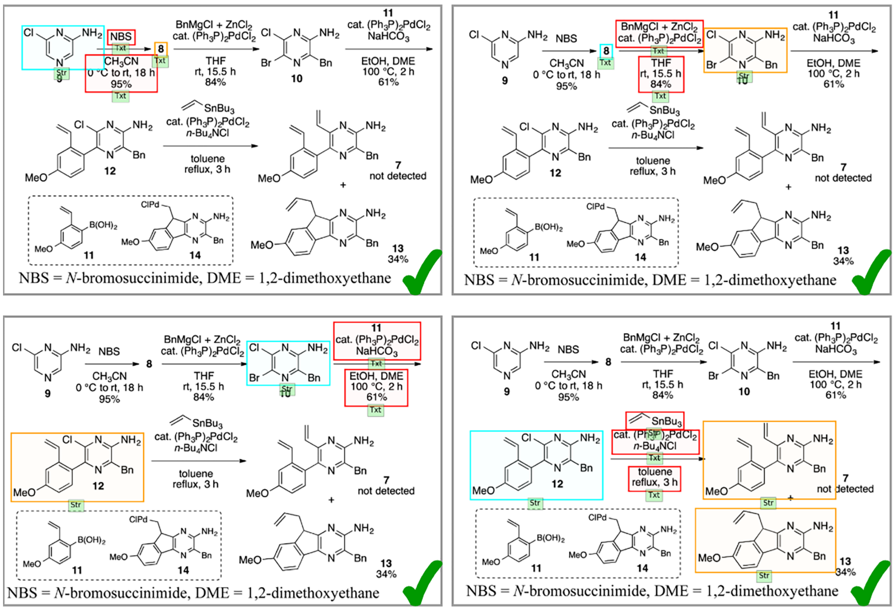

Fig. S2: **More visualization examples of the model's prediction on the reaction component identification task.** We showcase more complex examples of predicted reactions, each visualized in separate images. This is a multiple-line reaction image with four reactions.

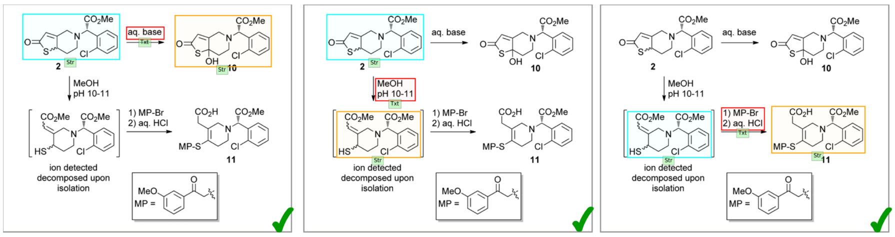

Fig. S3: **More visualization examples of the model's prediction on the reaction component identification task.** This is a branch reaction image with three reactions.

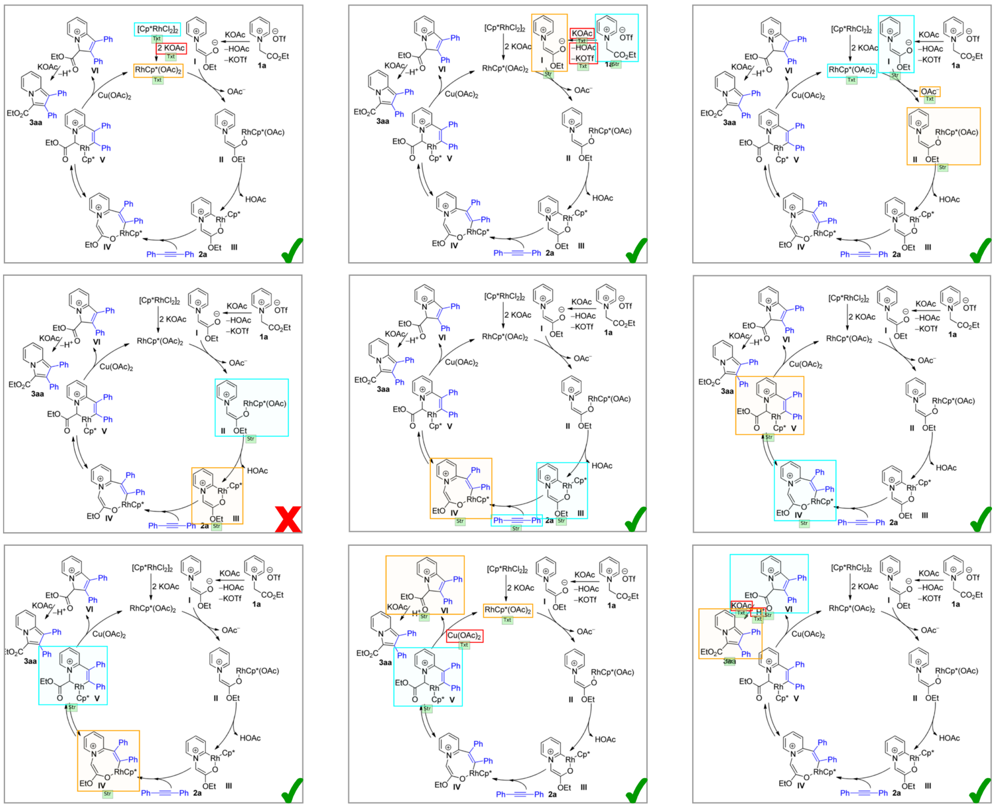

Fig. S4: **More visualization examples of the model's prediction on the reaction component identification task.** This is a cycle reaction image with nine reactions.

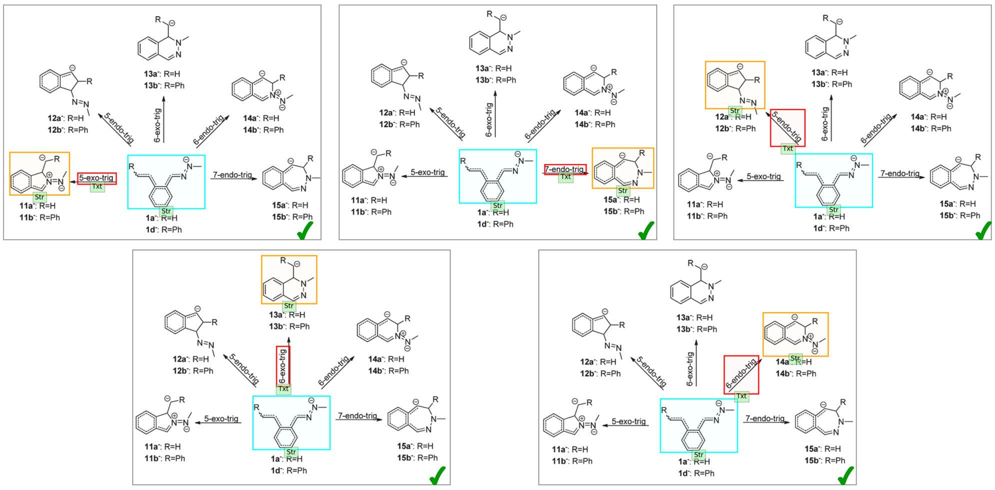

Fig. S5: **More visualization examples of the model's prediction on the reaction component identification task.** This is a radially branching reaction image with five reactions.

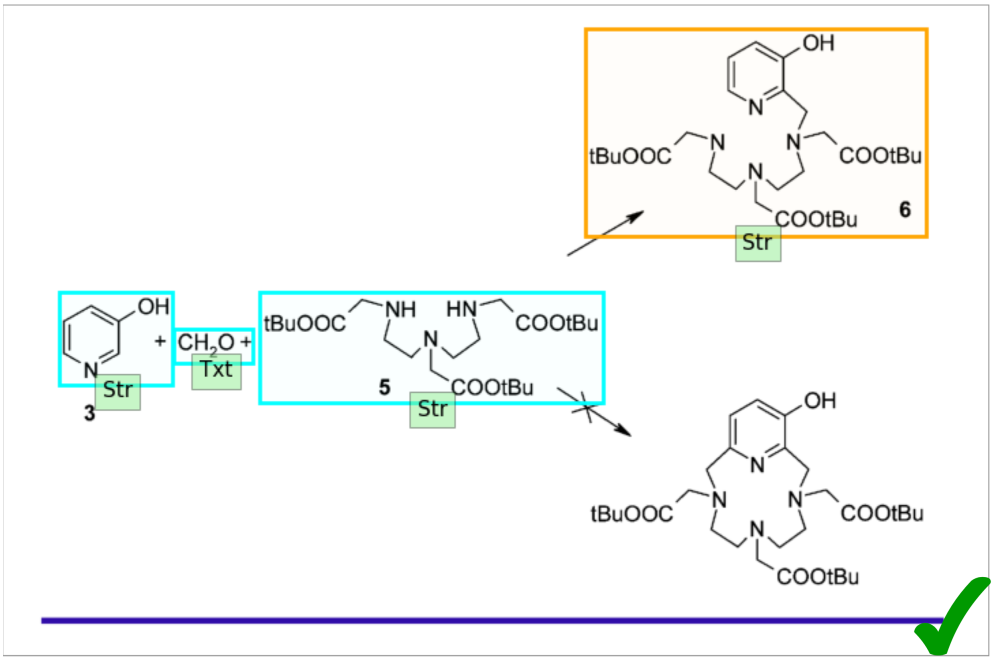

Fig. S6: **More visualization examples of the model's prediction on the reaction component identification task.** This is a branch reaction image with a cross (×).

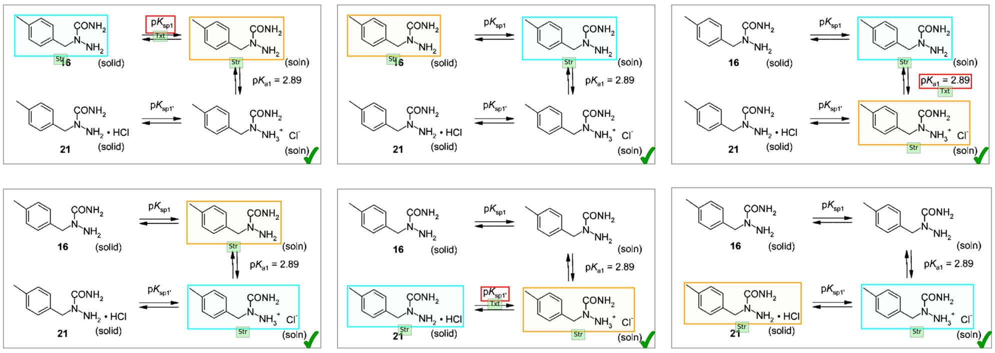

Fig. S7: **More visualization examples of the model's prediction on the reaction component identification task.** This is a branch reaction image involving three reversible reactions.

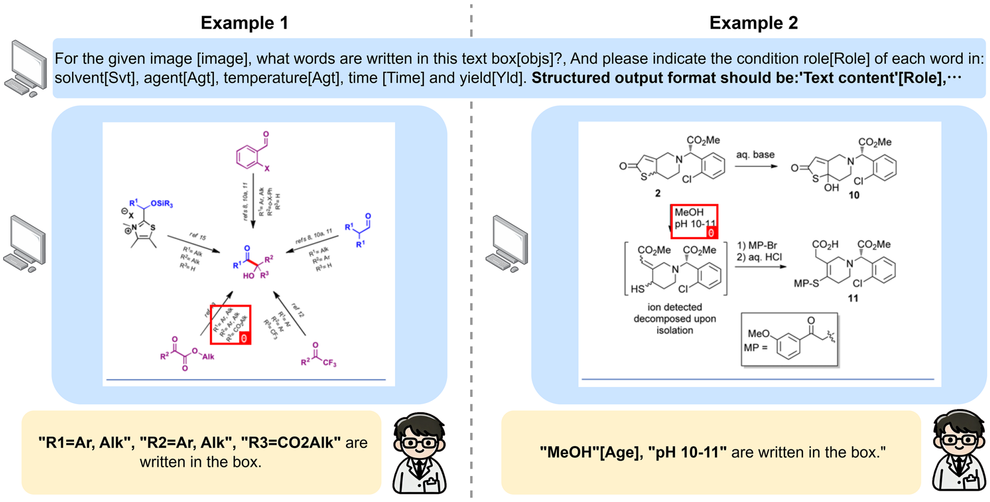

Fig. S8: **More visualization examples of the model's prediction on the reaction condition interpretation task.** Prediction 1 is a text region that contains several R-group information, and the text is tilted. Prediction 2 is a text region that contains "MeOH" and "pH 10-11".

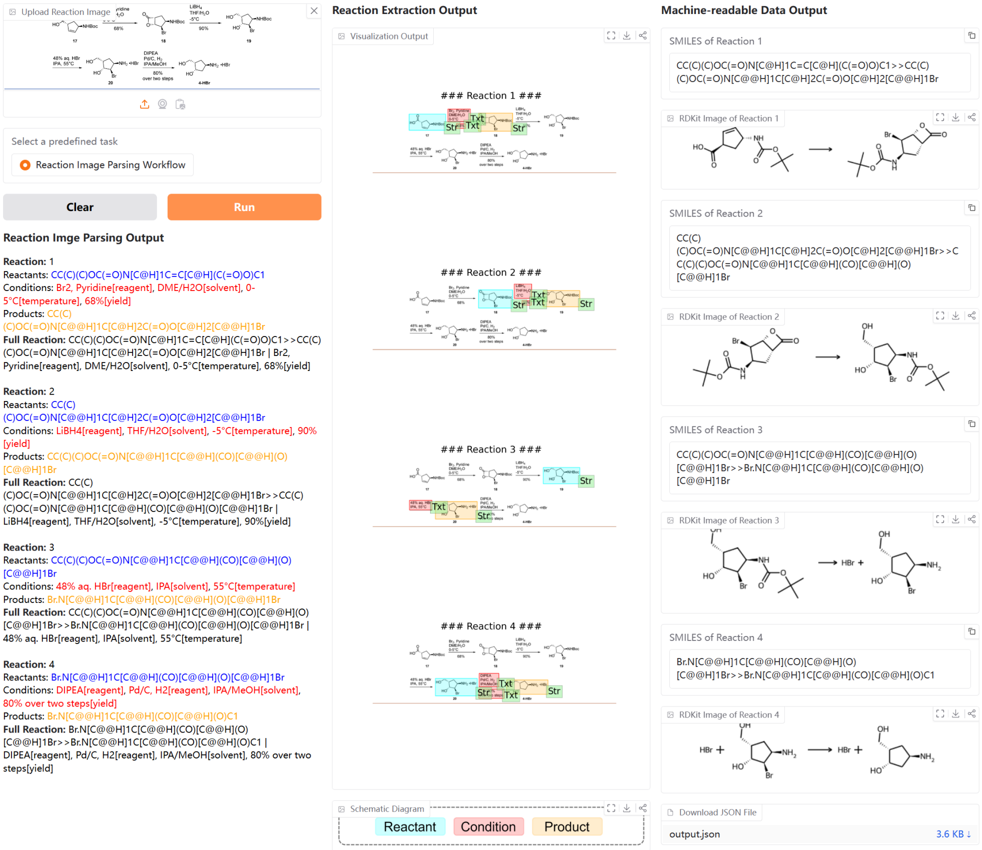

Fig. S9: **Example of a multiple-line reaction image (on the top left) that has been loaded into the RxnIM.web application.** We visualize the output of reaction component identification task and integrate the detailed results based on reaction image pasring output into machine readable formats such as reaction SMILES string and json format.

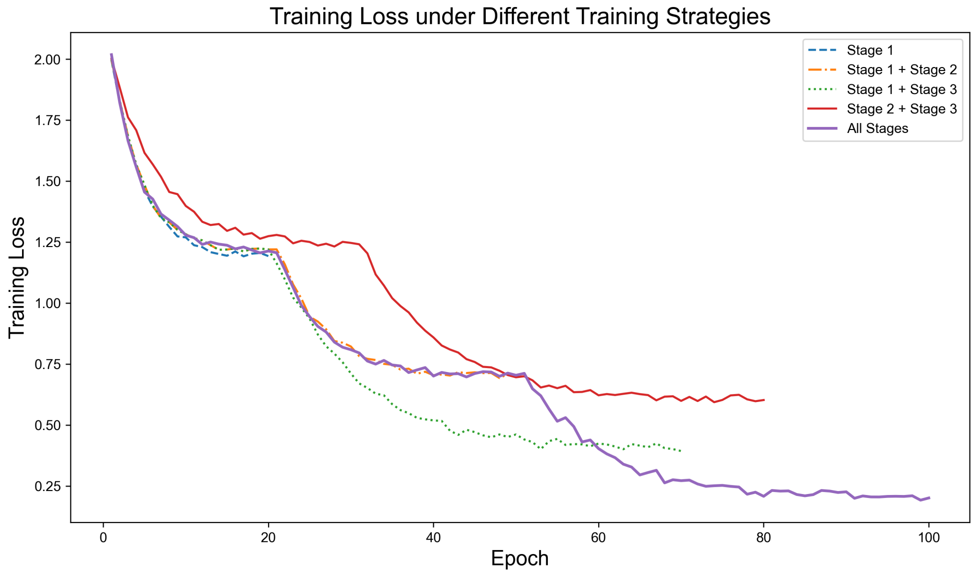

Fig. S10: **Training Loss under Different Training Strategies.**

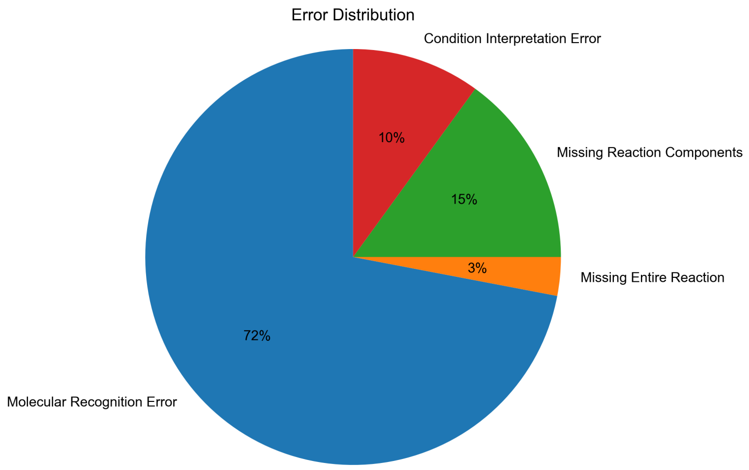

Fig. S11: **Distribution of error types in RxnIM predictions.**

References

[1] Qian, Y., Guo, J., Tu, Z., Coley, C.W., Barzilay, R.: Rxnscribe: A sequence generation model for reaction diagram parsing. Journal of Chemical Information and Modeling **63**(13), 4030–4041 (2023)

[2] Chen, T., Saxena, S., Li, L., Fleet, D.J., Hinton, G.: Pix2seq: A language modeling framework for object detection. arXiv preprint arXiv:2109.10852 (2021)

[3] Wang, W., Chen, Z., Chen, X., Wu, J., Zhu, X., Zeng, G., Luo, P., Lu, T., Zhou, J., Qiao, Y., et al.: Visionllm: Large language model is also an open-ended decoder for vision-centric tasks. Advances in Neural Information Processing Systems **36** (2024)

[4] He, K., Zhang, X., Ren, S., Sun, J.: Deep residual learning for image recognition. In: Proceedings of the IEEE Conference on Computer Vision and Pattern Recognition, pp. 770–778 (2016)

[5] Devlin, J., Chang, M.-W., Lee, K., Toutanova, K.: Bert: Pre-training of deep bidirectional transformers for language understanding. arXiv preprint arXiv:1810.04805 (2018)

[6] Vaswani, A., Shazeer, N., Parmar, N., Uszkoreit, J., Jones, L., Gomez, A.N., Kaiser, Ł., Polosukhin, I.: Attention is all you need. Advances in neural information processing systems **30** (2017)

[7] Chen, K., Zhang, Z., Zeng, W., Zhang, R., Zhu, F., Zhao, R.: Shikra: Unleashing multimodal llm's referential dialogue magic. arXiv preprint arXiv:2306.15195 (2023)

[8] Zhang, S., Sun, P., Chen, S., Xiao, M., Shao, W., Zhang, W., Liu, Y., Chen, K., Luo, P.: GPT4RoI: Instruction Tuning Large Language Model on Region-of-Interest (2024)

[9] Peng, Z., Wang, W., Dong, L., Hao, Y., Huang, S., Ma, S., Wei, F.: Kosmos-2: Grounding multimodal large language models to the world. arXiv preprint arXiv:2306.14824 (2023)

[10] Zhu, X., Su, W., Lu, L., Li, B., Wang, X., Dai, J.: Deformable detr: Deformable transformers for end-to-end object detection. arXiv preprint arXiv:2010.04159 (2020)

[11] Touvron, H., Lavril, T., Izacard, G., Martinet, X., Lachaux, M.-A., Lacroix, T., Rozière, B., Goyal, N., Hambro, E., Azhar, F., et al.: Llama: Open and efficient foundation language models. arXiv preprint arXiv:2302.13971 (2023)

[12] Touvron, H., Martin, L., Stone, K., Albert, P., Almahairi, A., Babaei, Y., Bashlykov, N., Batra, S., Bhargava, P., Bhosale, S., et al.: Llama 2: Open foundation

and fine-tuned chat models. arXiv preprint arXiv:2307.09288 (2023)

[13] Hurst, A., Lerer, A., Goucher, A.P., Perelman, A., Ramesh, A., Clark, A., Ostrow, A., Welihinda, A., Hayes, A., Radford, A., et al.: Gpt-4o system card. arXiv preprint arXiv:2410.21276 (2024)

[14] Wilary, D.M., Cole, J.M.: Reactiondataextractor 2.0: A deep learning approach for data extraction from chemical reaction schemes. Journal of Chemical Information and Modeling **63**(19), 6053–6067 (2023)
